# Habitat selection and ranges of tolerance: how do species differ beyond critical thresholds?

**DOI:** 10.1002/ece3.394

**Published:** 2012-10-09

**Authors:** Mary Ann Cunningham, Douglas H Johnson

**Affiliations:** 1 Department of Earth Science and Geography, Vassar CollegePoughkeepsie, New York, 12604, USA; 2 U.S. Geological Survey, Northern Prairie Wildlife Research CenterSt. Paul, Minnesota, 55108, USA

**Keywords:** Edge species, fragmentation, habitat preferences, habitat thresholds, interior species, landscape, LOESS, patch size, scale, tree cover

## Abstract

Sensitivity to habitat fragmentation often has been examined in terms of thresholds in landscape composition at which a species is likely to occur. Observed thresholds often have been low or absent, however, leaving much unexplained about habitat selection beyond initial thresholds of occurrence, even for species with strong habitat preferences. We examined responses to varying amounts of tree cover, a widely influential measure of habitat loss, for 40 woodland bird species in a mixed woodland/grassland landscape in eastern North Dakota, USA. We used LOESS smoothing to describe incidence for each species at three scales: within 200, 400, and 1200 m around sample locations. For the 200-m scale, we also calculated the most-preferred range of tree cover (within which at least half of observations were predicted to occur) for each species. Only 10 of 40 species had occurrence thresholds greater than about 10% tree cover. After initial occurrence, species showed three general patterns: some increased monotonically with tree cover; some increased up to an asymptote; some peaked at intermediate amounts of tree cover and then declined. These patterns approximate selection for interior woodlands and for edge-rich environments, but incidence plots provide greater detail in landscape-scale selection than do those categories. For most species, patterns persisted at larger scales, but for some, larger scales had distinctly different patterns than local scales. Preferred ranges of tree cover varied from <20% tree cover (common grackle, *Quiscalus quiscula*) to >60% (veery, *Catharus fuscescens*). We conclude that incidence patterns provide more information on habitat selection than do threshold measures for most species: in particular, they differentiate species preferring concentrated woodlands from those preferring mixed landscapes, and they show contrasting degrees of selectiveness. [Correction added on 16 October 2012, after first online publication: the Abstract section has been reworded].

## Introduction

**Figure 1:**
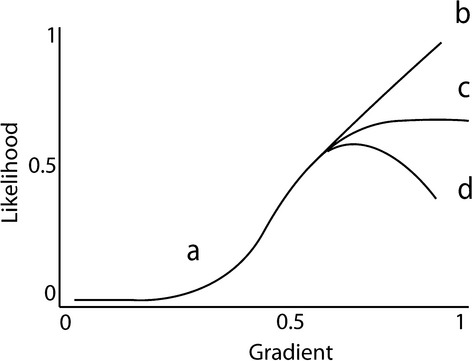
Possible variation in likelihood of species occurrence (Y) on a hypothetical environmental gradient (X). Thresholds of occurrence are frequently used to designate where a species first occurs (a). Beyond this threshold, likelihood of occurrence may increase monotonically (b), reach an asymptote (c), or decline after an initial increase (d). Those patterns of habitat selection may influence vulnerability to habitat loss or fragmentation.

Identifying degrees of sensitivity to habitat loss has been a central question for conservation of birds in fragmented and diminishing habitat. Sensitivity to landscape change often has been examined in terms of how strongly a species responds, in regression models, to variation in amount of a key land cover type, edge density, patch size, or other landscape-scale measures of fragmentation. Beyond strength and direction of response, identification of thresholds has been of particular interest for species conservation. Thresholds represent critical levels of environmental conditions, such as patch size or amount of a target land cover type, necessary for a species to occur (see Fahrig 2001; Hanski 2009; in some cases, thresholds have also be defined as points at which species abundance changes rapidly: Ficetola and Denoël 2009). Studies using segmented or piecewise regression have shown that models including thresholds can describe occurrence of some species better than models without thresholds (e.g. Betts et al. 2007, 2010; Zuckerberg and Porter 2011). Threshold measures also have theoretical value for issues such as metapopulation dynamics (Hanski 2009; Suding and Hobbs 2009). In general, thresholds are important indicators of the minimum value of a critical factor for species occurrence (Fig. 1, point “a”). But beyond this minimum critical value, species may respond in very different ways to increases environmental factors such as in habitat availability or patch size. Likelihood of occurrence may increase linearly, level off at an asymptote, or peak at an intermediate level (Fig. 1, lines b, c, and d). These contrasting responses beyond initial thresholds describe very different degrees of sensitivity to fragmentation in the landscape.

For many bird species, moreover, evidence of threshold responses to landscapes has sometimes been hard to identify, even for species observed to have strong patterns of habitat preference. For example, in a comparison of threshold models and linear models in woodland birds, Betts et al. (2010) found support for threshold models in 15 of 27 species examined. Many of these thresholds occurred at low levels of the preferred habitat in the nearby landscape, often less than 2%. Thus, while thresholds can improve on linear models of habitat response for some species, they may provide slight explanation for others, and statistically significant threshold values may describe little of the preferred habitat conditions for woodland birds.

Patterns of habitat selection beyond critical thresholds, such as multiple inflection points (Fig. 1) or narrowness of preferred ranges, could further explain vulnerability to landscape change. While thresholds can predict occurrence or extirpation of a species from an area, many conservation efforts are concerned with gradual population declines, before final extirpation occurs. Habitat selection beyond critical levels is important for understanding these gradual population declines that accompany habitat loss and fragmentation. Greater detail in describing patterns of habitat selection would be useful in identifying and comparing species' habitat preferences beyond thresholds of minimum tolerance, and in identifying species most vulnerable to habitat fragmentation.

For woodland birds, this question has long been addressed qualitatively in terms of interior and edge habitat selection. Sensitivity to landscape change has been evaluated by identifying which species are interior specialists, and how highly specialized these species are (Whitcomb et al. 1981; Robbins et al. 1989; Freemark and Collins 1992). The problem of identifying true interior specialists has remained an open question in studies of woodland bird ecology, with recent studies continuing to define which species require mature (often interior) woodlands, as compared to those that occupy early successional (often disturbed, fragmented, or edge) habitat (Streby et al. 2011; Chandler et al. 2012).

We examined habitat preferences beyond critical thresholds by plotting patterns of observed incidence for species on a gradient of landscape-scale tree cover. We chose amount of tree cover as our explanatory factor after comparing a variety of landscape-scale fragmentation metrics. Amount of tree cover in the landscape and edge density has shown strong effects for bird occurrence in previous studies (Andrén 1994; Parker et al. 2005; Desrochers et al. 2010; Cunningham and Johnson 2011). After establishing that this was a useful measure in our study area, we asked the following questions: (1) Do incidence patterns characterize habitat selection better than initial thresholds do? (2) Do species with similar patterns of habitat selection vary in narrowness of habitat selection? In addition, because landscape-scale responses frequently vary with scales of analysis, we asked: (3) Are patterns of selection evident principally at local scales, or can they also be detected at larger-landscape scales? Questions of habitat selection beyond the occurrence threshold are of broad interest because they offer greater clarity and precision in the question of species' sensitivity to habitat loss, and because they help broaden our view of critical measures that describe habitat requirements. Better definition of habitat responses can also aid in designing, managing, or evaluating habitat conservation efforts.

We examined these questions for 40 woodland bird species on a gradient of landscape fragmentation in a naturally fragmented, mixed woodland-grassland environment in southeastern North Dakota, USA. This analysis of 40 species shows a greater variety of responses than do many studies that compared fewer species. In this discussion we use the terms “habitat” and “habitat selection” to refer to landscape-scale selection for varying amounts or configuration of tree cover. In reality, species' habitat preferences vary among tree cover types and also may extend beyond tree cover to other types of vegetation. However this terminology and this approach to generalizing land cover are widely used (e.g. Freemark and Collins 1992; Parker et al. 2005; Radford et al. 2005) and they are better than other terms such as “landscape selection” at approximating the idea of selection for suitable resources in the landscape.

## Methods

Birds were surveyed using a belt transect design in both wooded and open areas in the Sheyenne National Grassland, in eastern North Dakota, USA, a study area that graded from open grassland to savanna to dense deciduous woodland. The area consisted of 28,000 ha of public lands as well as adjoining farmland, scattered woodlands, and pasture. Woodlands were composed mainly of oak (*Quercus macrocarpa*), aspen (*Populus tremuloides*), and other mixed deciduous tree cover. Grasslands comprised a diverse mix of perennial grasses and forbs, with areas of low-growing shrubs (largely western snowberry, *Symphoricarpos occidentalis*) and scattered areas of taller willow shrubs (*Salix* spp.). Manske (1980) and Seiler and Barker (1985) have provided detailed descriptions of the area's vegetation. Martin and Svingen (2010) and Cunningham et al. (2006) have documented the region's avifauna in detail. Birds were counted along belt transects (Stewart and Kantrud 1972; Igl and Johnson 1997) 2–6 km in length. One observer walked these transects slowly (1 km/hour), recording all birds seen or heard within 50 m on either side. Birds flying over the transect were not included in analysis. A global positioning system (GPS) unit was used to divide transects into 100-m segments and to record bird counts by these segments, which could later be geo-referenced to land-cover data. Bird counts were done between 0.5 hours before sunrise and 4 hours after sunrise, in winds <20 km/hr and temperatures between 5 and 25°C, between late May and early July from 2002 to 2005. The same observer conducted surveys in all years. All sites were visited once because we expected greater added information from a larger sample of the landscape than from repeat visits, because our aim was to record presence/absence for common species during peak breading season, and because the sample size was large. Details on sample design can be found in Cunningham and Johnson (2006).

Indicated breeding pairs were counted. If sexes were alike, the number of singing males was counted. If no individuals were singing, then the number of visually observed individuals was halved and rounded up to derive indicated pairs. For brown-headed cowbirds, females and males were recorded separately, and we report females. The final data set included 3,261 transect segments from 4 years. Preliminary analysis indicated that interactions between year and other variables were minimal, and that including a year effect to account for variation in abundance among years had little effect on our results (Appendix S1), so we pooled years in subsequent analysis.

### Land cover data and landscape measures

Measures of tree cover in the landscape have been widely shown to have good predictive ability for woodland bird responses to landscape fragmentation, and this class of landcover has been used widely in studying habitat selection in the past (e.g. Parker et al. 2005; Desrochers et al. 2010; Schipper et al. 2011). Therefore we used tree cover as our focal landcover class in spatial analysis. We calculated class metrics for tree cover around each 100-m transect segment as follows: Tree cover was digitized in ArcGIS (ESRI 2004) from georeferenced 1-m resolution digital aerial photographs. Because of the relatively high resolution of the images, we had a minimum mapping unit of 10 m^2^, and isolated trees were digitized individually. Digitized tree cover data were converted to raster format with a cell size of 5 m.

We used FRAGSTS (McGarigal et al. 2002) and the FragStatsBatch utility in ArcGIS 9.2 (ESRI 2004; Mitchell 2007) to calculate landscape composition and configuration variables within radii of 200, 400, 800, 1200, and 1600 m around each 100-m segment. In order to select a fragmentation metric for analysis, we initially calculated the following measures for tree cover in the landscape: percentage tree cover in the landscape, edge density (m/ha), amount of core area ( >50 m from an edge), patch cohesion (a measure of how aggregated wooded patches are), largest patch index (proportion of the landscape comprising the largest single patch), and mean patch size. For variable definitions, see McGarigal et al. (2002).To test patch size as an explanatory variable, we also calculated in ArcGIS the size of the largest patch that occurred on or adjacent to a segment. We assessed similarity among landscape variables and among scales by calculating Pearson correlation coefficients.

### Statistical analysis and variable selection

Our aim was to compare responses of many species to landscape composition, rather than to maximize explanation of individual species' occurrences on multiple simultaneous gradients. For comparing many species or scales, it can be useful to focus on one widely influential explanatory factor. To identify a most influential variable, we used both the model-ranking and weighting approach of Burnham and Anderson (2002) and logistic regression to compare species' strength of response to the metrics listed above. Details of this analysis and results are given in Appendix S1.

Among fragmentation measures, percentage tree cover was the best explanatory variable for most species with strongest responses to landscape variables (e.g. great-crested flycatcher, scarlet tanager, rose-breasted grosbeak; scientific names are listed in Table 1). Percentage tree cover and edge density were best for 11 species each, followed by largest patch index (4 species), and patch cohesion (3 species). For species that were relatively poorly explained by all variables, differences among variables were slight (e.g. mourning dove, eastern kingbird, song sparrow). The overall average *R*^2^ value was highest for tree cover. These findings were consistent at 5 different scales (Appendix S1). Among the fragmentation measures, then, percentage tree cover and edge density were similar in their influence on species. Because edge density is scale-dependent, in that it is influenced by scale and grain of analysis (Wu et al. 2002), we used percentage tree cover as our landscape descriptor for subsequent analysis.

**Table 1:** Species names and number of transect segments on which species occurred

Species	*N*
Mourning Dove (*Zenaida macroura*)	323
Yellow-bellied Sapsucker (*Sphyrapicus varius*)	161
Hairy Woodpecker (*Picoides villosus*)	40
Northern Flicker (*Colaptes auratus*)	88
Eastern Wood-Pewee (*Contopus virens*)	231
Willow Flycatcher (*Empidonax traillii*)	44
Least Flycatcher (*Empidonax minimus*)	338
Great Crested Flycatcher (*Myiarchus crinitus*)	53
Eastern Kingbird (*Tyrannus tyrannus*)	265
Yellow-throated Vireo (*Vireo flavifrons*)	47
Warbling Vireo (*Vireo gilvus*)	133
Red-eyed Vireo (*Vireo olivaceus*)	145
Blue Jay (*Cyanocitta cristata*)	75
Tree Swallow (*Tachycineta bicolor*)	95
Black-capped Chickadee (*Poecile atricapillus*)	62
White-breasted Nuthatch (*Sitta carolinensis*)	70
House Wren (*Troglodytes aedon*)	361
Eastern Bluebird (*Sialia sialis*)	78
Veery (*Catharus fuscescens*)	21
American Robin (*Turdus migratorius*)	130
Gray Catbird (*Dumetella carolinensis*)	161
Brown Thrasher (*Toxostoma rufum*)	71
European Starling (*Sturnus vulgaris*)	29
Cedar Waxwing (*Bombycilla cedrorum*)	67
Yellow Warbler (*Dendroica petechia*)	205
Black-and-white Warbler (*Mniotilta varia*)	40
Ovenbird (*Seiurus aurocapillus*)	118
Scarlet Tanager (*Piranga olivacea*)	62
Chipping Sparrow (*Spizella passerina*)	38
Field Sparrow (*Spizella pusilla*)	277
Vesper Sparrow *(Pooecetes gramineus)*	300
Lark Sparrow (*Chondestes grammacus*)	127
Song Sparrow (*Melospiza melodia*)	33
Rose-breasted Grosbeak (*Pheucticus ludovicianus*)	22
Indigo Bunting (*Passerina cyanea*)	25
American Goldfinch (*Carduelis tristis*)	275
Baltimore Oriole (*Icterus galbula*)	196
Orchard Oriole (*Icterus spurius*)	64
Common Grackle (*Quiscalus quiscula*)	95
Brown-headed Cowbird (*Molothrus ater*)	92
All segments	3261

Amount of tree cover in the landscape was correlated with other fragmentation metrics. At the 200-m scale, percentage tree cover was strongly and positively correlated with edge density (Pearson's *r* = 0.82) and largest patch index (*r* = 0.81). Percentage tree cover was moderately correlated with patch cohesion (*r* = 0.58), percentage core area (*r* = 0.50), and maximum patch size on a segment (*r* = 0.68). Correlations were also strong between measures of tree cover calculated at different scales: percentage tree cover within 200 m was strongly correlated with that within 400 m (*r* = 0.93) and within 1200 m (*r* = 0.72).

### Independence of observations

Our original data included contiguous 100-m transect segments. Data from these segments were not statistically independent, but in final analyses we used all observations for two reasons (detailed in Appendix S1). First, independence of observations is important for evaluating the significance of parametric tests, which underestimate error with non-independent data, and thus overestimate the significance of results in hypothesis testing (Diniz-Filho et al. 2003). In the current study we show *patterns* in the occurrence of birds, rather than testing hypotheses about occurrence, and thus our argument does not hinge on significance. Non-independent data can be suitable when hypothesis testing is not the goal of a study (Pan 2001; Diniz-Filho et al. 2003). Second, any dependencies in our data were more than offset by the large number of data points (3,261 segments). Another way to consider this is that while data from these segments were not statistically independent, they represented an average of multiple subsamples evaluated in preliminary analysis. To test this idea and the importance of independence on our conclusions, we compared the output of the processes above using (1) the whole data set and (2) five different subsets of the data, in which all segments were separated by at least 400 m. Our results indicated that independence did not alter our findings for the preferred range of tree cover for a species, either for species that occurred with relatively even frequency across the years or those that occurred less frequently in some years. Results of these comparisons are shown in Appendix S1. We also examined the effects of differences among years and concluded that they had little effect on our results (see Appendix S1).

### Incidence plots

We plotted likelihood of occurrence for woodland species on a gradient of percentage tree cover, from 0 to 75% (the maximum percentage that occurred at the 200 m scale). To calculate likelihood of occurrence from the presence/absence data collected in the field, we sorted all 100-m segments by percentage tree cover within 200 m, and then grouped the sorted segments into even-sized groups (42 groups of 76, and one of 69). For each group, we calculated the observed incidence (frequency of occurrence) for each species. We also calculated the average percentage tree cover in each group. Using values of these group averages, then we then were able to plot continuous (rather than binary) values for frequency of occurrence against percentage tree cover. We fitted a curve to each plot using SAS PROC LOESS, a locally weighted regression and scatterplot smoothing method that calculated predicted probabilities of occurrence based on local regression (Cleveland and Devlin 1988; SAS Institute 1996). We used a smoothing parameter of 0.5. Preliminary analysis with varying group sizes and smoothing parameters indicated that these parameters had little influence on the width of tree ranges identified from plots (see below). We calculated 95% confidence limits using the method of Cohen (1999), after confirming that residuals were approximately normally distributed. The resulting plots showed the estimated frequency of a species' occurrence along a gradient of increasing tree cover.

### Scale comparisons and favored ranges

We performed these procedures first using percentage tree cover calculated within 200 m of transect segments. To evaluate whether patterns persisted at larger scales, we repeated incidence plots using percentage tree cover within radii of 400 and 1200 m around transect segments.

To calculate most favored ranges on the gradient of tree cover from incidence plots, we identified the minimum range of X (tree cover) for which at least 50% of observations were predicted to occur in the smoothed curve. We calculate this range by deriving the minimum range of X that represented half of the area under the curve of the LOESS plot.

## Results

**Figure 2:**
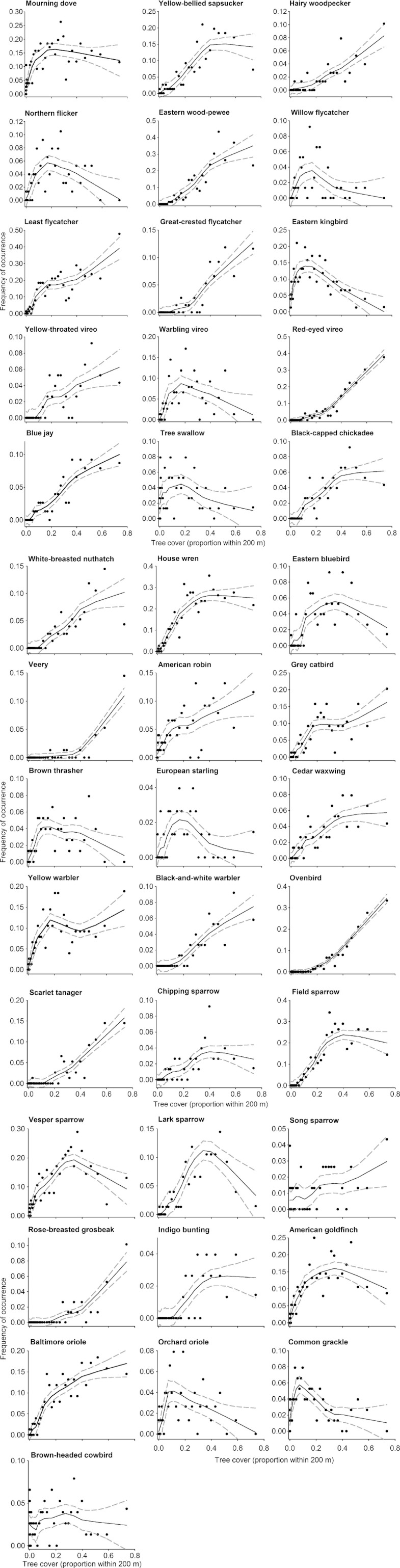
Incidence patterns showing occurrence in response to proportion tree cover within 200 m for 40 species. Dots represent groups of 76 observations; lines show smoothed patterns and their 95% confidence intervals.

We analyzed 40 species that were associated with woodlands and that were recorded on more than 20 transect segments. When incidence was plotted on a gradient of tree cover, a variety of responses were evident, ranging from monotonic increases (preference for wooded landscapes) to narrow peaks (preferences for mixed landscapes: Fig. 2). Among these responses, four general patterns emerged. The most tree-dependent species were absent or nearly absent up to thresholds of 20–40% tree cover, after which their frequency of occurrence increased proportionally with tree cover (e.g. veery, ovenbird, rose-breasted grosbeak). Other species increased proportionally with tree cover but sometimes occurred with very slight tree cover (e.g. hairy woodpecker, red-eyed vireo, white-breasted nuthatch). For these species, there was little evidence of a threshold, even though there was strong selection for woodland-rich environments in many of these species. A third general group of species showed an initial response to tree cover followed by little response to increasing cover beyond 20–40% (e.g. mourning dove, house wren, field sparrow). A fourth group peaked with moderate amounts of tree cover and became less common in areas with more than 40–50% tree cover (e.g. northern flicker, vesper sparrow, lark sparrow). Only one species, brown-headed cowbird, occurred with roughly similar frequencies at all levels of tree cover.

### Preferred ranges

**Figure 3:**
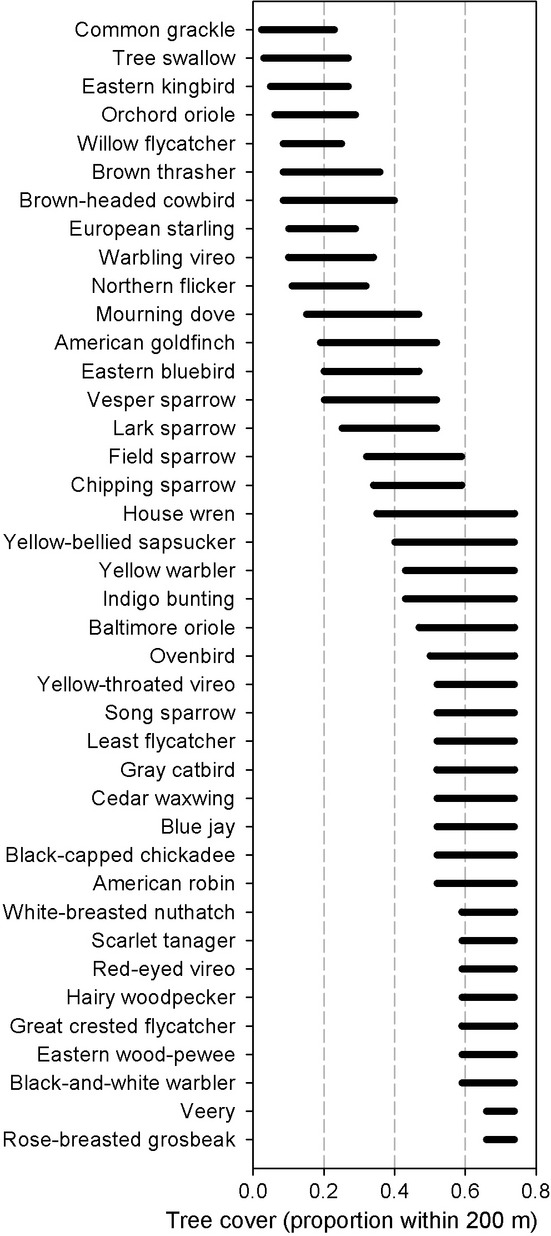
Proportion of tree cover at which species were most likely to occur. Ranges represent peaks of LOESS curves, i.e. the narrowest range for which half of observations occurred.

Species with similar patterns of habitat selection exhibited differences in narrowness of habitat selection (Fig. 3). Some occurred most frequently in landscapes with less than 30% tree cover (e.g. common grackle, tree swallow, orchard oriole, eastern kingbird, and willow flycatcher). Other species were most common in landscapes with more than 60% tree cover (e.g. rose-breasted grosbeak, veery). Some species had relatively narrow ranges in mostly wooded landscapes (e.g. ovenbird, rose-breasted grosbeak, veery); others had relatively narrow peaks in more open landscapes (compare ranges, for example, of northern flicker and chipping sparrow to those of vesper sparrow and American goldfinch). Other species were likely to occur under a broad range of conditions (e.g. brown-headed cowbird, mourning dove, yellow warbler). In our study area, the maximum percentage tree cover average for groups in incidence plots (at 200 m) was 75%. It is likely that some species would be most abundant in more heavily wooded landscapes if they were available.

### Scale comparisons

**Figure 4:**
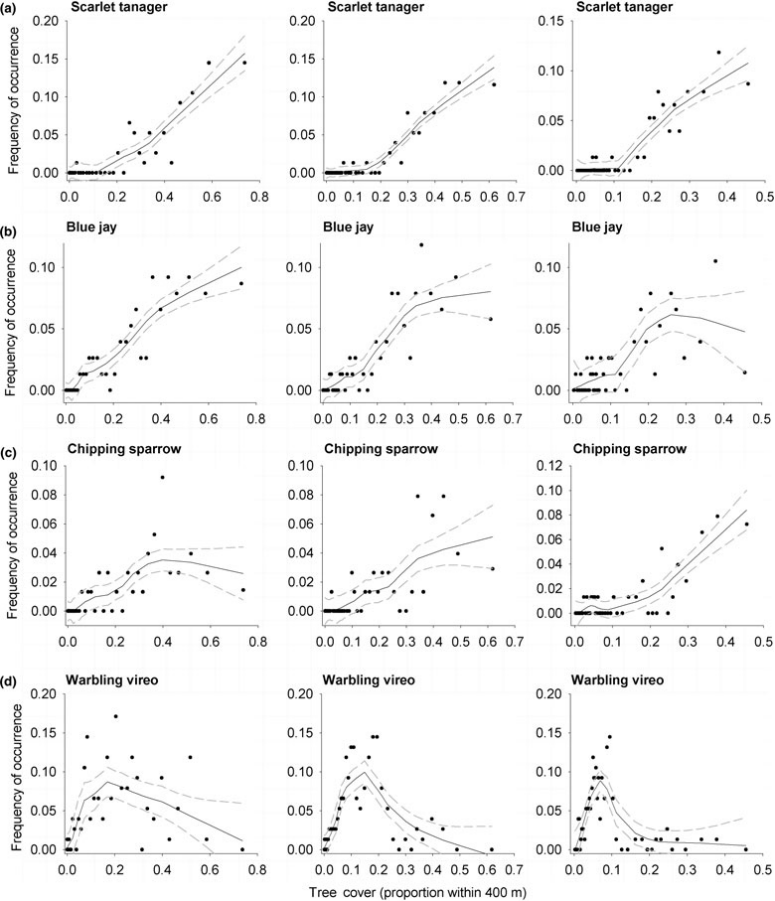
Scale variation in response to percentage tree cover (200, 400, and 1200-m radii around 100-m transect segments). Examples are shown for species that have similar patterns at different scales (a), contrasting responses at smaller and larger scales (b, c), and similar patterns but narrower selection at larger scales (d).

For most species, patterns of selection were evident at larger-landscape scales, as well as at local scales (Fig. 4). When examined at larger scales (larger radii), some species showed persistent preferences for the maximum available tree cover and can be considered interior sensitive at large scales (Fig. 4a). Others preferred interior conditions at proximate scales but were generally indifferent to tree cover at larger scales (Fig. 4b). Several species that preferred edges when evaluated at proximate scales preferred wooded landscapes at larger scales (Fig. 4c). Finally, some of the species that avoided heavily wooded conditions at the proximate scale had still stronger avoidance of abundant tree cover at large scales (Fig. 4d). Plots for all species at 200, 400, and 1200-m scales are available in Appendix S2. Landscape measures at different scales were strongly correlated, so we cannot say conclusively that the larger scale effects were not simply an artifact of local-scale responses for many species (although incidence patterns were still evident at larger scales). But for some species with contrasting patterns of response at local scales and larger scales (e.g. chipping sparrow), selectiveness was evident even at larger scales.

## Discussion

Incidence plots showed strongly contrasting patterns beyond initial thresholds of occurrence. Thresholds in occurrence above approximately 10% tree cover were evident for 10 of 40 species (great-crested flycatcher, yellow-throated vireo, red-eyed vireo, white-breasted nuthatch, veery, black-and-white warbler, ovenbird, scarlet tanager, rose-breasted grosbeak, indigo bunting). For the remainder of species, we found thresholds to be slight or absent. Some species that showed strong preferences for abundant woodlands still occurred at low levels of tree cover, though with reduced frequency (e.g. hairy woodpecker, Fig. 2). While those low-tree cover areas may not be the core of a nesting territory, they composed part of the area in which these birds were foraging or singing. For a majority of our species, then, threshold measure provided little information about degrees of selectiveness at the landscape scale. Patterns of occurrence with increasing amounts of tree cover gave substantial information about habitat selection, however. Some species strongly selected for more-wooded landscapes, some were selective only at low levels of tree cover (e.g. <20%), and some preferred mixed landscapes. Incidence plots are helpful in visualizing these contrasting responses. Quantifying favored ranges from incidence plots further helps in distinguishing species that prefer abundant woodlands (which were correlated with less-fragmented woodlands) from those that tolerate a wide range of fragmentation conditions.

Previous studies have explored threshold responses to measures such as minimum patch size (Whitcomb et al. 1981; Robbins et al. 1989), or amount of tree cover in an area (Betts et al. 2010; Desrochers et al. 2010). Threshold identification often has been elusive for temperate-zone migratory birds, however, owing in part to the wide range of habitat tolerance in many species. Variation in habitat use even by habitat specialists has been noted previously (Villard 1998).

Responses to a single environmental variable reflect only one of the many factors that may influence habitat selection, such as vegetation structure, food availability, nest sites, or predator abundance. For studies seeking to maximize explanation of habitat selection, multivariable modeling approaches can incorporate many environmental influences more completely than single-variable incidence plots can. But for studies aiming to compare findings for multiple species and multiple scales, responses to a single widely influential variable are informative. An incidence plot approach also is useful for comparing responses in different study areas or contrasting landscape contexts: Patterns found in our study area may differ from those in areas that are dominated by woodland habitat, for example. Similar studies in contrasting environments would help to further characterize the full range of a species' responses to fragmentation and to landscape composition.

### Alternatives to the single-variable tree cover approach

Incidence plots could be based on any landscape metric, such as edge density, cohesion, or amount of core habitat, but we focused on amount of tree cover because it explained well those species with strong responses to any landscape variable (Table 1, Fig. 2). Amount of tree cover also was correlated with other measures of fragmentation. For some species that showed an asymptote or a peak in the middle range of tree cover, edge density or cohesion gave marginally stronger explanation–this is not surprising since these are generally considered edge species. Even for species with relatively slight responses to landscape metrics, however, such as mourning dove or vesper sparrow (Fig. 2), incidence plots using tree cover showed distinct patterns of response. Many scholars focusing on landscape metrics have been wary of amount of tree cover as a primary explanatory factor, perhaps because other measures such as patch size, amount of core area, or connectivity measures are better supported by theory (Haila 2002). For example, the equilibrium theory of island biogeography (MacArthur and Wilson 1963) has served as a foundational approach to understanding fragmented terrestrial landscapes, establishing the idea that habitat patches and their proximity are critical explanatory factors. However, patchy landscapes are not island archipelagos, and temperate-zone birds do not colonize patches as species colonize islands (Norton et al. 2000; Brotons et al. 2003). Empirically, amount of tree cover has often provided better explanation than patch size or patch proximity measures (Andrén 1994; Villard 1998; Parker et al. 2005; Desrochers et al. 2010).

Amount of tree cover has the additional advantage that it is not scale dependent, as are measures such as edge density, which varies with the grain or resolution of landcover data (Wu et al. 2002). In addition, this measure is easily reproducible using a variety of digitizing or data collection methods, is insensitive to scales of analysis, and is relatively insensitive to degrees of detail in digitizing. Core area, edge density, and many patch measures, in contrast, vary with the scale and resolution with which land cover data are interpreted or analyzed. Amount of tree cover (or amount of habitat more generally) is also easy to visualize and understand, relative to many landscape metrics. In addition, measures of fragmentation and habitat loss frequently covary (Parker et al. 2005), making amount of cover frequently representative of extent of fragmentation as well as habitat availability.

### Interior and edge species and sensitivity to fragmentation

Selection of landscape conditions beyond initial occurrence thresholds often has been characterized in terms of interior and edge habitat selection. These categorical designations have been used to distinguish species that prefer edge-rich environments from those that may occur occasionally in low-tree cover areas but that strongly prefer abundant tree cover (Whitcomb et al. 1981; Robbins et al. 1989). The species with linear responses in our incidence plots have been identified previously as interior species or interior-edge species, or alternatively as mature-forest species, and species selecting mixed habitat have been identified as edge species or as early-successional species (Whitcomb et al. 1981; Robbins et al. 1989; Imbeau et al. 2003; Streby et al. 2011; Chandler et al. 2012). The argument has been made that interior/edge and early/late successional habitat designations overlap, as edge-rich environments in a forested region are often early successional areas, as in the case of clear-cut patches (Imbeau et al. 2003; Schlossberg and King 2008). These ideas coincide in many study areas, but they are functionally distinct. Edge versus interior distinctions refer to spatial configuration of habitat, while the late versus early successional characterizations refer to the temporal stage of habitat. The savanna landscape in our study area consisted mainly of mixed woodlands and open grasslands, rather than the woodlands and regenerating clear cuts examined in many studies of woodland birds. While the landscape mosaic of our study area shifts over time, edges and open areas are not necessarily early successional for this biome. Consequently, interior and edge designations are more useful for study areas like ours than early successional and mature forest designations.

The incidence patterns shown here reflect responses to amount of tree cover, rather than the amount of “interior” or “edge” habitat per se, but they do suggest some considerations for how those categories might be understood. First, there was considerable overlap among groups. Although general groupings can be defined along the lines shown in Fig. 1, species examined here exhibited a continuum of incidence patterns, from peaks (preferences for 30–60% tree cover) to asymptotes to linear increases in occurrence with increasing tree cover. It is challenging to group such patterns into simple categorical designations such as interior and edge species. Moreover, within this continuum, species with similar incidence patterns varied in narrowness of habitat preference. For example, both the veery and red-eyed vireo had strong preferences for high concentrations of habitat, but the veery had a narrower range of most-preferred habitat conditions (Fig. 3). The vesper sparrow, meanwhile, had twice the range of northern flicker, another edge species. Not all interior species are alike, and edge species also can vary in how much edge they prefer.

Second, detection of “interior” habitat selection can depend on the scale of analysis. It has been observed frequently that species respond to environmental factors at different scales (e.g. Desrochers et al. 2010). For most species, responses were similar at local scales and at larger landscape scales, presumably reflecting the similarity in tree cover at the three scales analyzed, but a number of species had strong responses to amount of tree cover mainly at local scales (e.g. hairy woodpecker, great-crested flycatcher; Appendix S2). A few had more distinct responses at larger scales (e.g. chipping sparrow, Fig. 3). The warbling vireo, meanwhile, had a wide tolerance of mixed habitat conditions at a local scale but occurred only in areas that were mostly open at a larger scale. The chipping sparrow occupied a variety of mixed landscapes at the local scale, but only in areas that were mostly wooded at the larger scale. Thus detection of habitat response patterns may require multiple-scale analysis, as well as clear indications of the larger landscape context within which patterns of habitat selection are found (Andrén 1994).

Third, there is considerable variation in habitat selection within a species. Incidence plots show that the habitat selection is variable and probabilistic: for species with strong preferences for abundant tree cover, incidence increased gradually, not abruptly, after first occurrence. Thus species designated as “interior” species may not occur only in interior habitat. Similarly, “edge” species occurred most frequently in mixed landscapes, but they also occurred occasionally in dominantly wooded areas. This range of tolerance presumably reflects frequent use of sub-optimal habitat, for example by younger or less competitive individuals. But mixed habitat may also have advantages, even for interior species. Vitz and Rodewald (2011) showed that fledgling ovenbirds and worm-eating warblers (*Helmitheros vermivorum*) use habitat with more dense lower-level vegetation and a more open canopy than adults use for nesting. Similarly, Streby et al. (2011) found that rose-breasted grosbeak, red-eyed vireo, and scarlet tanager nested both in mature, interior forest and near early successional edges, and Chandler et al. (2012) found that seven of nine mature-forest specialists were most abundant in early-successional habitat after fledging. Variation in habitat selection may reflect a more-diverse use of resources than has often been acknowledged, in addition to occupancy of sub-optimal habitat.

Fourth, amount of total habitat in an area is a useful predictor of the occurrence of interior species. In comparison to tree cover (and edge density, which was closely correlated to amount of tree cover in this study area), amount of core area and patch size provided relatively modest explanation for most species, with the exception of the veery (Appendix S1). It may be that species requiring true core habitat were absent from our landscape or were not numerous enough to analyze. However, in a comparable analysis done in a forested study area in New York, total forest area was also more informative than core area, although the two were strongly correlated (Cunningham and Johnson 2011). This is not to say that contiguous, unfragmented forest is not important. Fragmentation is often a snowballing process, in which initial road building, forest clearing, or suburban development lead to further and accelerating losses of habitat. Moreover, temperate-zone migratory songbirds may be more tolerant of fragmentation than many other less-mobile organisms. Amphibians and understory plants such as spring ephemerals, for example, may be more strongly affected by forest fragmentation than birds, and for these organisms, core woodland habitat may be critical.

### Implications for conservation

Most conservation activities involve managing landscapes for multiple species. In such situations, it is useful to understand similarities and differences in responses among groups of species. Those similarities and differences can be easiest to evaluate in terms of responses to a common landscape factor, such as abundance of habitat. Threshold measures in response to habitat abundance provide critical information about tolerance of fragmentation and change in landscape composition, but incidence patterns are more informative for identifying preferred habitat conditions as well as narrowness of tolerance. Thresholds were evident for a few species, but nearly all species showed distinct selection patterns with regard to amount of tree cover. The gradual changes evident in incidence plots represent real variation around expected habitat selection patterns, which can be considerable, even in species designated as interiors specialists. Differences in breadth of tolerance and in preferred habitat composition can help explain population abundance and trends in an area, as landscape composition changes, and as the availability of landscapes with preferred habitat composition increases or decreases.

## Supporting Information

**Appendix S1.** Methods: Landscape variable selection, independence, and year effects.

**Appendix S2.** Results: Incidence plots for all species at 200, 400, and 1200 m scales.
