# Supplementary material for: Habitat selection and ranges of tolerance: how do species differ beyond critical thresholds?
Source: Ecol Evol. 2012 Oct 9;2(11):2815–28. doi: 10.1002/ece3.394 (PMC3501633; doi:10.1002/ece3.394)
Supplement: Supplementary file 1 [file ece30002-2815-SD1.doc]

**Appendix S1**

**Methods: variable selection, independence, and year effects**

In this paper we have taken a number of steps that bear further explanation. In this appendix we describe several aspects of our methods in greater detail than we can do in the paper itself. The following sections thus document the decision process involvedin establishing the variables used in analysis.

**1. Selecting an explanatory landscape variable**

Our aim was to compare responses of many species, rather than to maximize explanation of individual species’ occurrences, and for this comparison we needed to identify one widely-influential factor in the landscape. We expected that we could identify an overall-best variable using variable weights derived from model ranking using Akaike’s Information Criterion (AIC; Burnham and Anderson 2002). Preliminary analysis indicated that the strongest explanatory variables overall were edge density, percentage tree cover, and patch cohesion (hereafter “cohesion”). We included these three variables plus maximum patch size (frequently considered important) in model ranking (using PROC GENMOD, link LOGIT in SAS; SAS Institute 1996). Models included each variable individually, all pairs, a 4-variable (complete) model, and a null model. The 4-variable model was best or competitive for all species. Other combined models were competitive for most species (edge density + cohesion for 20 species; percentage tree cover + cohesion for 18 species). Single-variable models were rarely competitive. After model ranking, Akaike weights were assigned to each model, representing the weight of evidence for that model among the models considered. The Akaike weight for a model was proportional to exp(-0.5 *delta AIC), and the sum of all model weights was 1.0. Weights for individual variables were then calculated by summing the weights of all models in which a variable appeared (Burnham and Anderson 2002). This measure helps to indicate relative influence of individual variables. A variable that occurred in all competitive models had a weight of 1.

Akaike weights for variables turned out to be inconclusive in identifying a single-best variable. While weights account for relative rank among models, they do not account for amount of explanation, which in some cases was poor. For example, all models explained the occurrence of the brown-headed cowbird (*Molothrus ater*) poorly, and no variables had significant coefficient estimates in regression. Because models performed similarly, however, all models had equal weights, all variables contributed equally to the “best” models, and all variables had weights of 1. These weights indicated that all variables performed as well as the very best variable, even though that variable provided little explanation. This weighting was not differentiated from the best variables for a species that was explained well, and for which variable weights were strongly differentiated, such as the red-eyed vireo (*Vireo olivaceus*). Thus model ranking and variable weights were not unambiguous for the purpose of identifying an explanatory measure.

Because the AIC approach was not unambiguous for our purpose, we compared regression coefficients from single-variable logistic regression models in order to select a single broadly-informative variable. We regressed each species against each of the 4 landscape variables that were used in the AIC routine. For comparison, we also included the other landscape variables noted above. We used quadratic models (probability of occurrence = 1/(1 + exp(-(β _0_ + β _1_ x + β _2_ x ^2^ ))), because quadratic models had stronger responses than linear models for most variables and species. We did this at 5 different landscape scales, ranging from 200-m to 1600-m radius around the 100-m segments. Overall, coefficients were greatest at the 200-m scale, so we used this as the primary scale for analysis.

Percentage tree cover and edge density were equivalent in their influence on species. Because of the relatively high values for the best-explained species, percentage tree cover had the highest overall average R^2^ values. This finding was consistent at 5 different scales (Table 1). Logistic regression results using fragmentation variables calculated within 200 m for each species are shown in figure 2. In addition to percentage tree cover and edge density, two correlated measures of cohesion and largest patch index (LPI) were strong for a number of species (e.g., for least flycatcher, white-breasted nuthatch, veery, field sparrow: Fig. 1). For those species where any variable had an R^2^ > 0.2, the best variables were percentage tree cover (best for 7 species), followed by edge density (2 species), cohesion, and largest patch index (each best for 1 species). Species with intermediate strength of response (any R^2^ > 0.1 but all R^2^ < 0.2), the best single variables were percentage tree cover and edge density (best for 11 species each), followed by largest patch index (4 species), and cohesion (3 species). For species that were relatively poorly explained by all variables, differences among variables were slight (e.g., mourning dove, eastern kingbird, song sparrow). Because edge density is scale-dependent, in that it is influenced by scale and grain of analysis (Wu et al. 2002), we used percentage tree cover as our landscape descriptor for subsequent analysis.

Table 1. Average R^2^ value for each variable for all species at each of 5 landscape scales, using quadratic models. Strongest measures at each scale are bolded.

|  |  |  | Scale (m) | | |  |  |
| --- | --- | --- | --- | --- | --- | --- | --- |
| Variable | 200 | 400 | | 800 | 1200 | | 1600 |
| Pct tree cover | **0.14** | **0.12** | | **0.10** | **0.08** | | **0.08** |
| Edge density | 0.13 | 0.11 | | 0.09 | **0.08** | | 0.07 |
| Cohesion | 0.13 | 0.10 | | 0.07 | 0.06 | | 0.05 |
| Largest patch index | 0.11 | 0.08 | | 0.05 | 0.05 | | 0.05 |
| Core area | 0.04 | 0.02 | | 0.01 | 0.01 | | 0.01 |
| Mean patch size | 0.06 | 0.06 | | 0.05 | 0.05 | | 0.05 |

Amount of tree cover was correlated with other measures of fragmentation. Percentage tree cover was strongly and positively correlated with edge density (Pearson’s r = 0.82, using tree cover calculated within 200 m) and largest patch index (r = 0.81). Percentage tree cover was moderately correlated with cohesion (r = 0.58), percentage core area (r = 0.50), and maximum patch size on a segment (r = 0.68). Correlations were also strong between measures of tree cover calculated at different scales: percentage tree cover within 200 m was strongly correlated with that within 400 m (r = 0.93) and within 1200 m (r = 0.72).

Figure 1. Magnitude of logistic regression coefficient (R^2^) values for quadratic models predicting presence or absence of a species. Circle diameter is proportional to the R^2^ value for landscape metrics representing tree cover within 200 m of a segment.

**2. Independence**

A possible concern in our analysis is that we did not account for possible dependence between variables on adjacent segments of transects. Certainly a transect segment will be intrinsically more similar to an adjacent segment than to one some distance away. Moreover, adjacent segments have nearly identical surrounding landscapes, so they are non-independent in that manner, as well. Hence non-independence of adjacent segments is nearly certain. Is non-independence of concern here?

Statistical independence is invoked in a variety of circumstances during analysis, such as when computing probabilities under specified hypotheses. Independence allows one to compute the probability of a series of events as the product of the probabilities of the individual events. But independence is not always a requisite. Consider an example in which one wishes to estimate the average height of male students in high school classes. Suppose one of the classes includes a set of identical twins. Clearly their heights are not independent. One could eliminate that non-independence by (randomly) choosing one of the two students and excluding his height from the calculation. However, if heights of identical twins differ from non-twins, then elimination of one of the twins results in a biased estimate of average height. Hence the need to ascertain whether or not independence of observations is necessary or even desirable in some applications.

A commonly used approach to deal with non-independence is to use only a fraction of the data set, say every fifth segment in our case, so that segments can more realistically be considered independent. Suppose we did that, using only segments 1, 6, 11, 16, etc. We could estimate the curves and other outputs we show based on this fraction of independent data. Then we could repeat the process, next using segments 2, 7, 12, 17, etc. Ultimately we would obtain five different curves, each of which is based on a set of (more-or-less) independent observations.

We used this approach and compared incidence plots (LOESS curves) from five subsamples of our data to the entire data set. Subsamples were extracte by taking every fifth transect segment, as noted above. Thus subsamples 1 includes segments 1, 6, 11, 16, etc.; subsamples 2 inludes segements 2, 7, 12, 17,… and so on. The results are plotted below for the five subsamples (in color), each of one-fifth of the data, as well as the LOESS curve based on the entire data set (in black: Fig. 2). Which of the five curves should be used? Each has equal credibility. Alternatively, we could somehow average the curves, to obtain a single curve that reflects all of the observations. But this is fundamentally the same as using all of the data initially, which is what we were trying to avoid.

We repeated this process for all 16 of the species with at least 20 observations in each subset (Fig. 3). Three conclusions are evident from these plots: 1) the curve based on the entire data set is in fact representative of the overall pattern manifested by the five individual curves; 2) the curve based on the entire data set is, as would be expected, smoother than curves based on partial data sets; and, most importantly, 3) a single curve based on partial data (note curve 5 in the American robin example) may not be representative of the patterns shown by the majority of the curves. To the latter point, curve 5 suggests that the occurrence of American robins peaks at about 10 percent tree cover and is indifferent as tree cover ranges from 25 percent to 70 percent. All other curves show an increasing likelihood of occurrence with increasing tree cover. Using only a fraction of the data would definitely be wasteful of information, resulting in unjustified jaggedness, and could well be misleading, depending on how representative the selection fraction of the data are. It would also be unnecessary, because independence of the observations is not a requirement for such summaries of data.


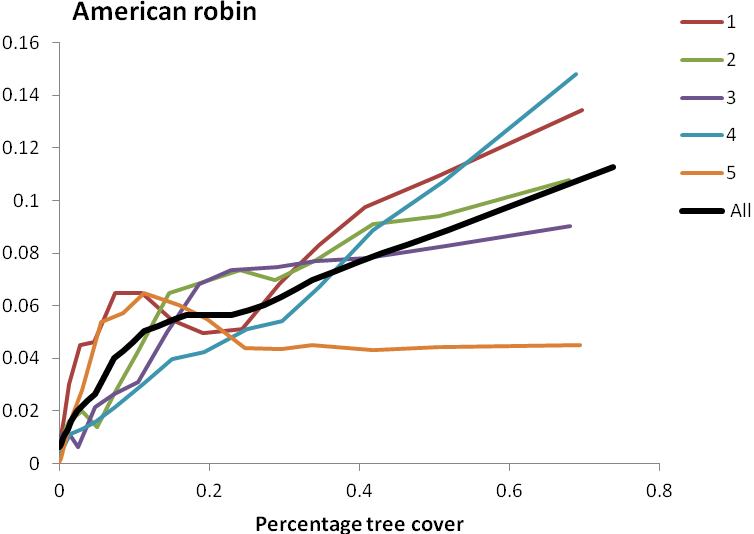


Figure 2 Incidence plots calculated using subsamples of the data and all data for American robin.


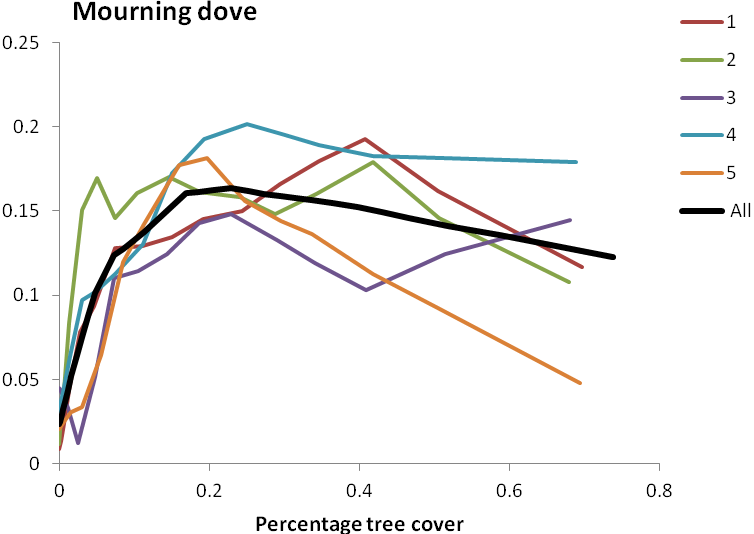

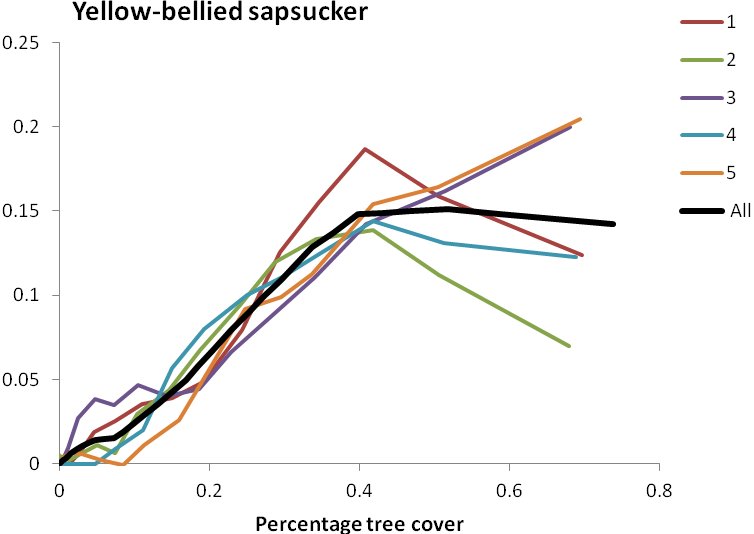

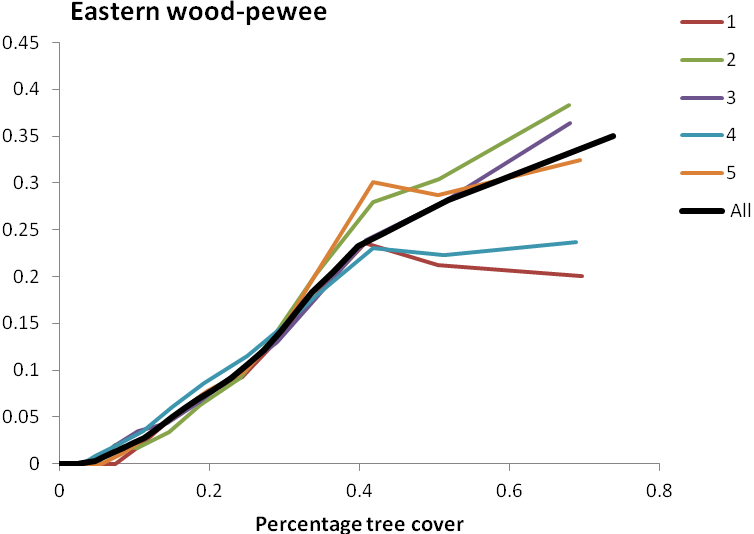

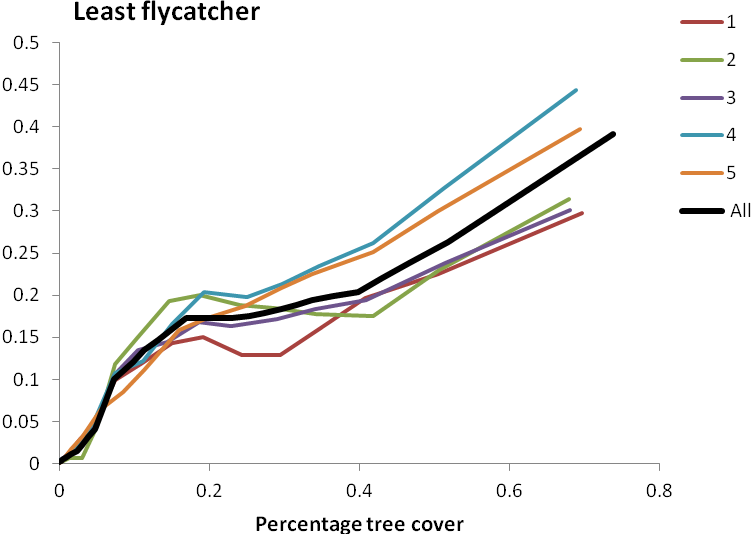

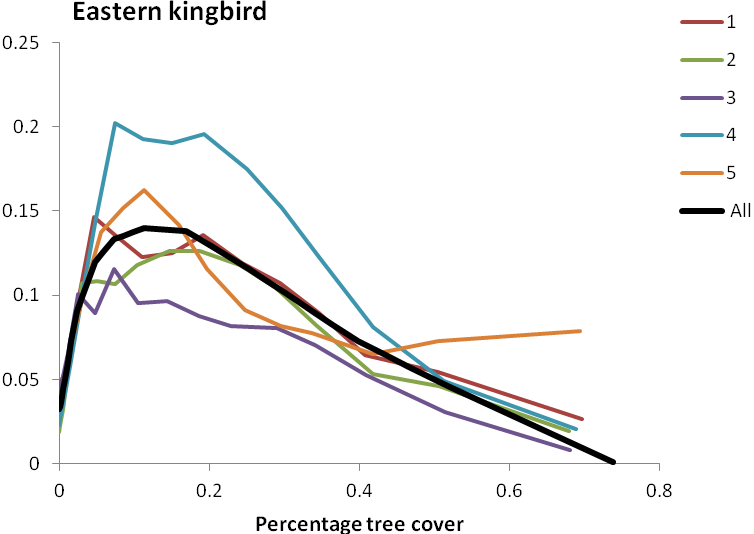

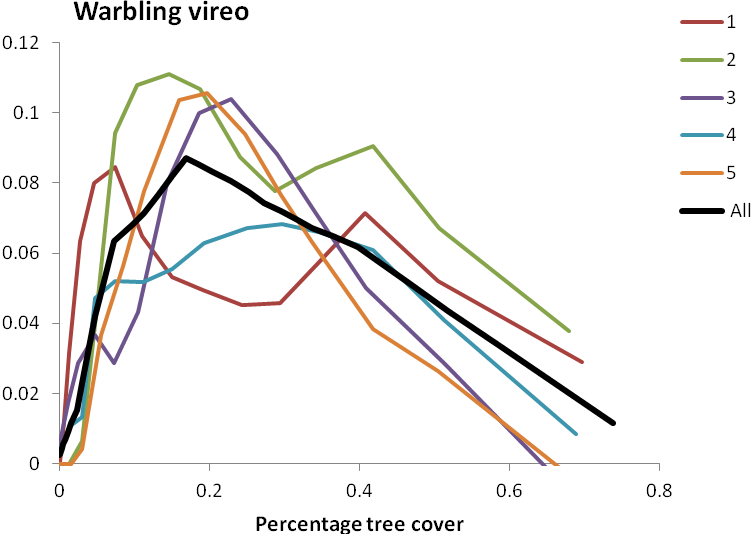

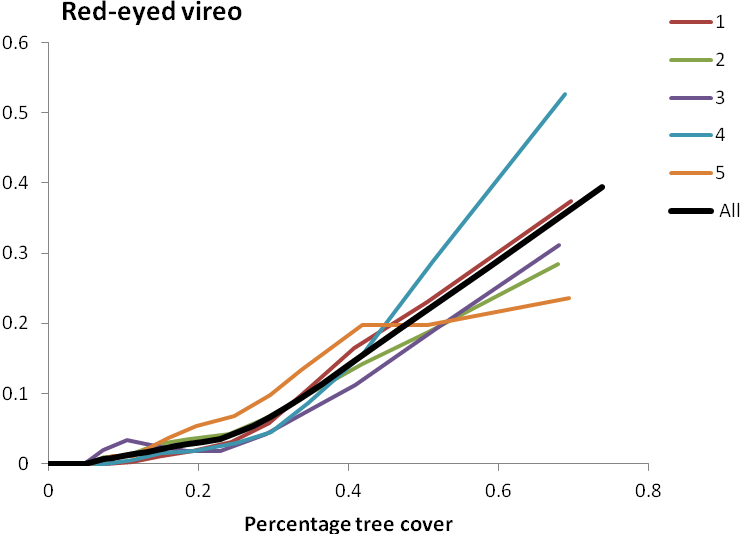

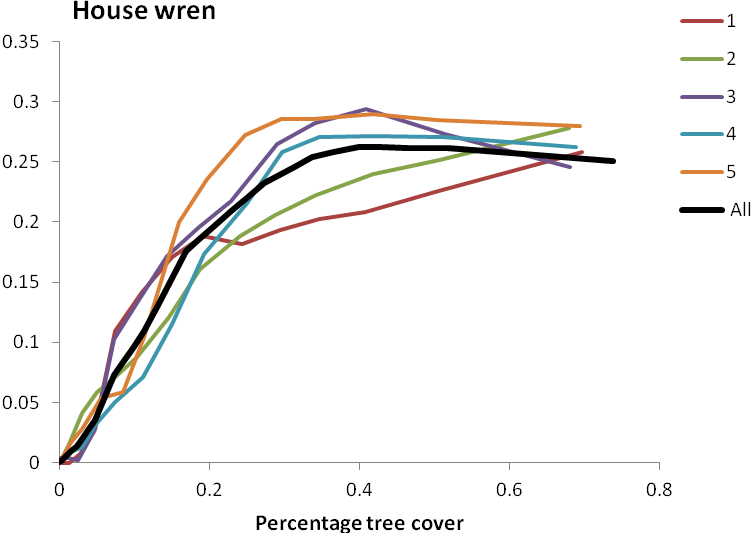


Figure 3a Incidence plots calculated using subsamples of the data and all data for 8 of 16 species.


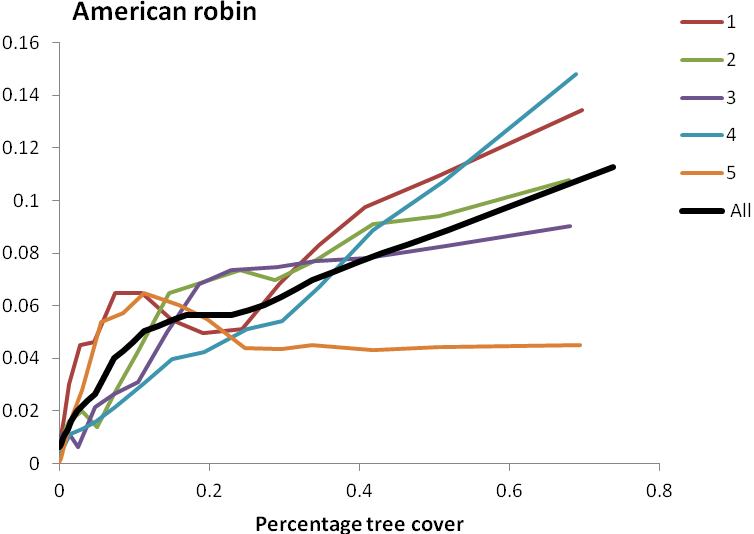

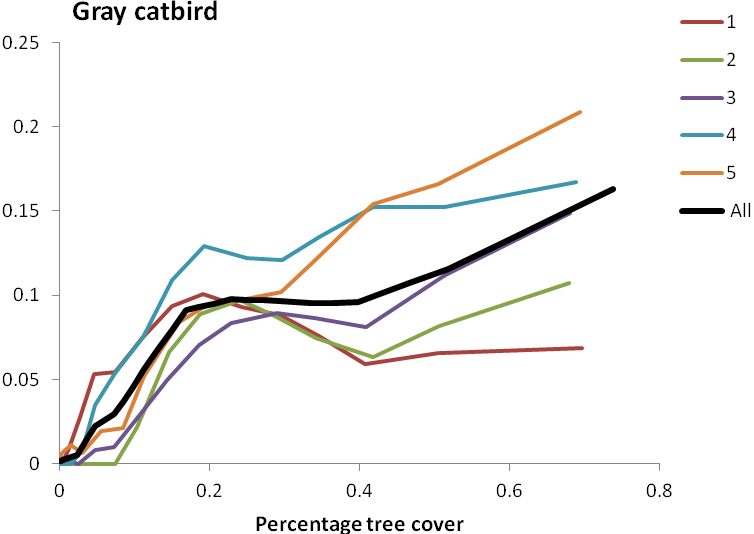

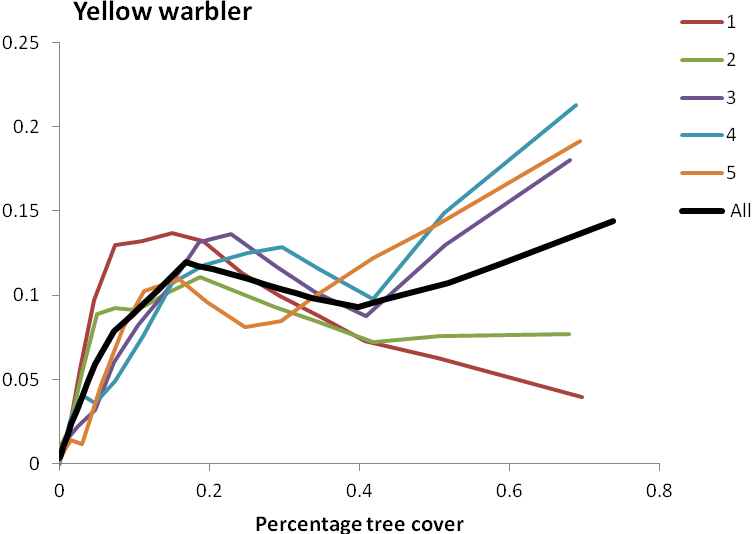

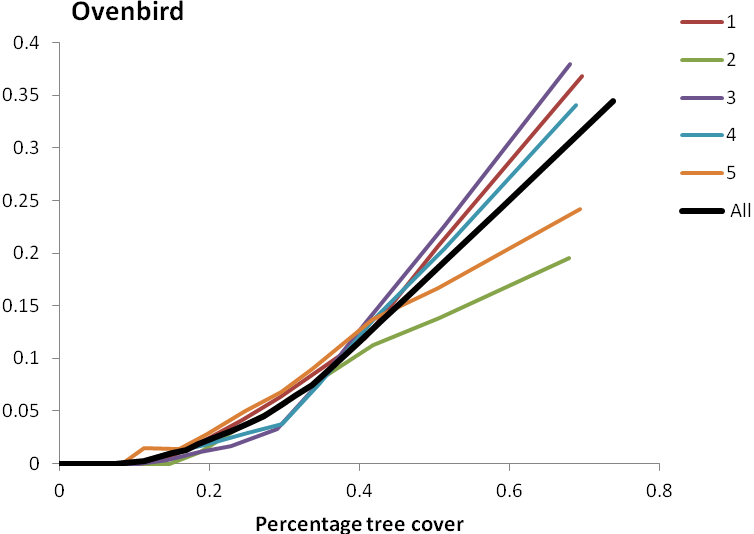

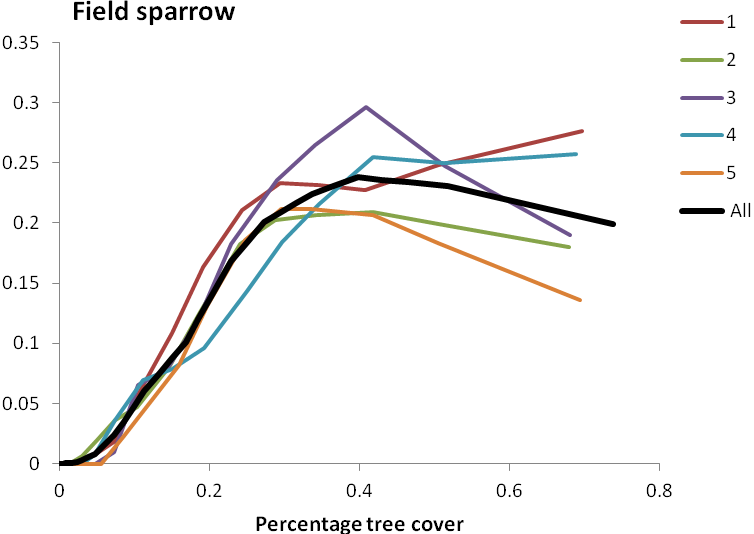

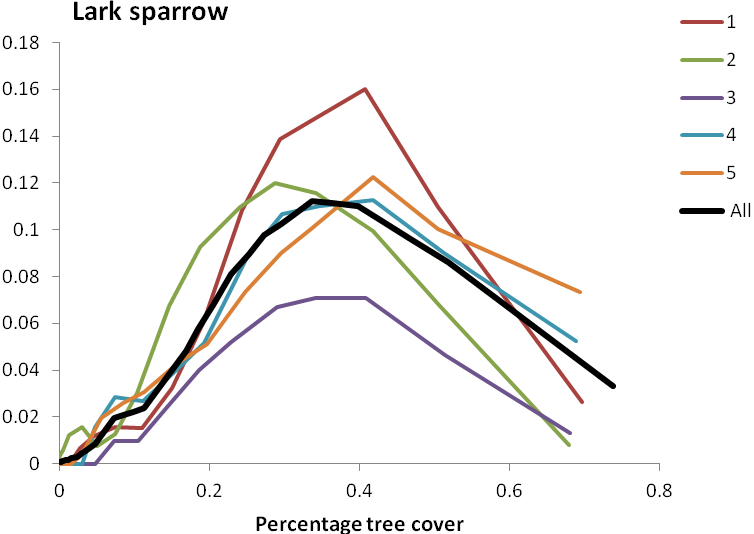

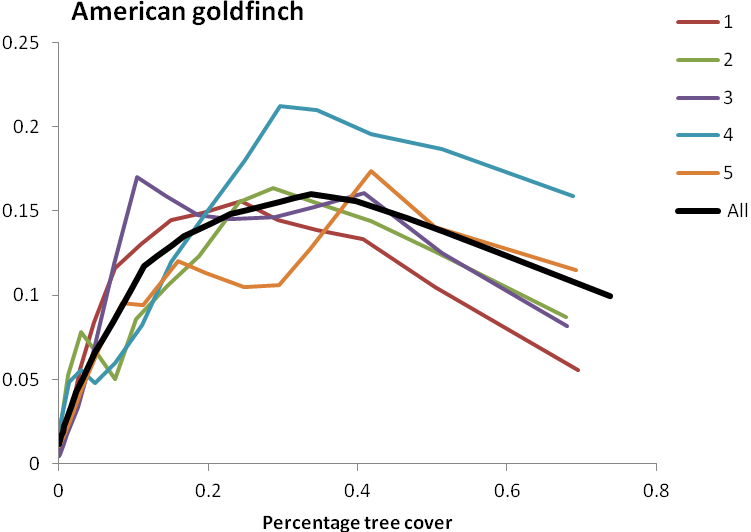

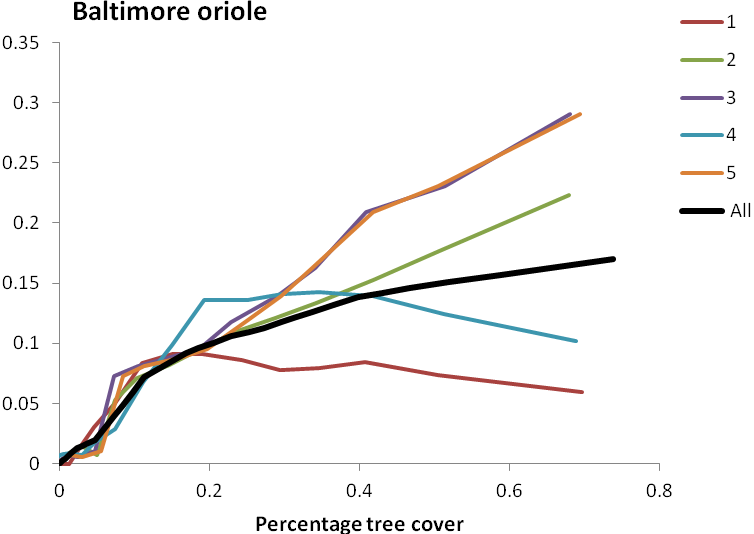


Figure 3b Incidence plots calculated using subsamples of the data and all data for 8 of 16 species.

**3. Year effects**

Because the abundance and occupancy rates of birds can vary dramatically among years, it is important to consider that variation when estimating preferences for habitat types. As an extreme example, suppose that in one year tree-favoring scarlet tanagers were absent, and if that was the only year that many heavily wooded transects were surveyed, one would obtain mostly zero occupancy values in most of the heavily wooded segments and conclude that the species avoids trees. Numerically, suppose scarlet tanagers were observed on 5 of 500 segments (overall occurrence rate = 0.01) in one year. Next suppose the species was much more common and widely distributed the following year, occurring on 20 of 400 segments (overall occurrence rate = 0.04). We likely would have less-favorable segments occupied than in the previous year, so any preference or selection for certain segments would be less evident.

We can reduce that potentially biasing effect by dividing the presence or absence value (1 or 0) for each segment by the overall occurrence rate in that year. Hence an occurrence value in the first year would be divided by 0.01, producing values of 100 or 0 for each segment. In the second year occurrence rates would be divided by 0.04, yielding values of 25 or 0. This adjustment scales upward occurrence values in the first year, when the species was less ubiquitous.

Mathematically, suppose the frequency of occurrence of a particular species in year *t* on segment *j* is *f_jt_* (= 0 or 1). Then the overall occurrence rate of that species in year *t* is the number of segments on which the species was recorded, divided by the number of segments surveyed in year *t*: *f._t_* = ∑*_i_* *f_jt_* /*N_t_*. Then scaled occupancy values *f’_jt_* = *f_jt_* / *f._t_* will account for annual variation in occupancy when used to develop incidence plots.

We compared incidence plots developed from both standard and scaled occupancy values (*f_jt_* and *f’_jt_*, respectively) for species with relatively even distributions among years (Fig. 4) and for species with uneven distributions among years (Fig. 5). Generally the profiles were very similar, regardless of whether standard or scaled occupancy values were used. Thus, for simplicity we present results based on the more familiar 1/0 occupancy values.


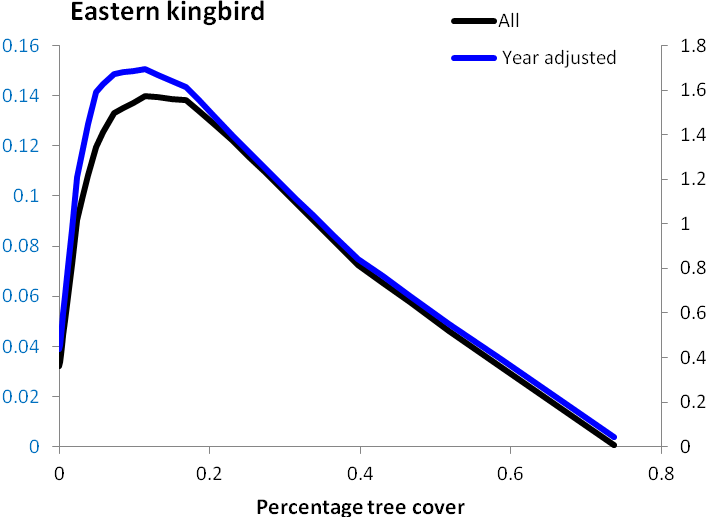

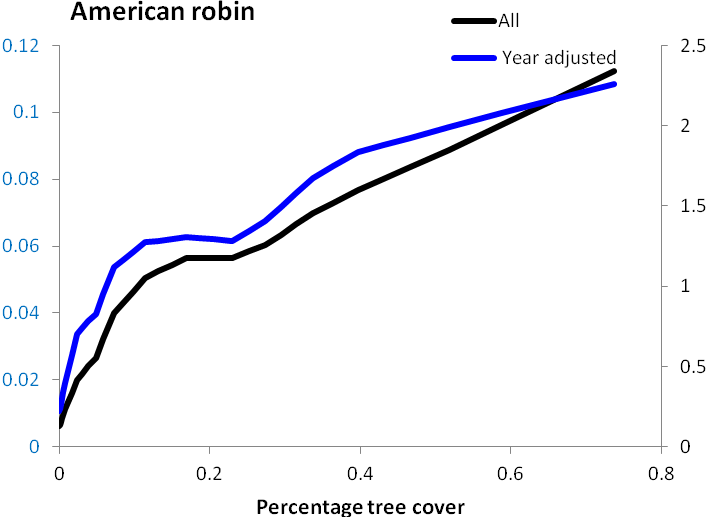

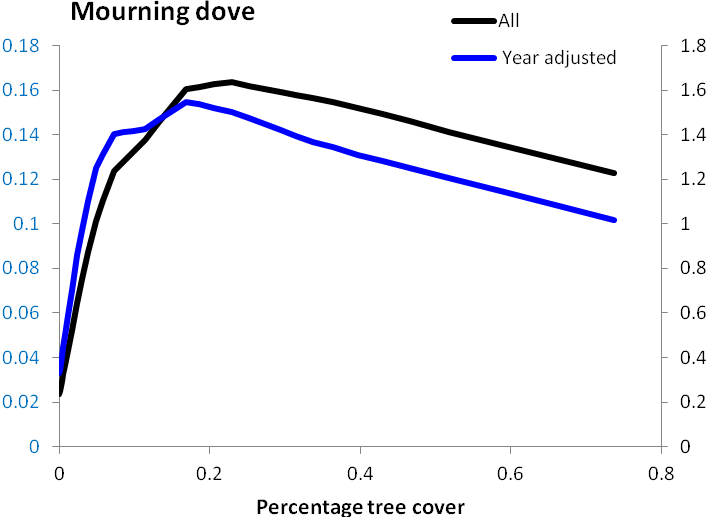

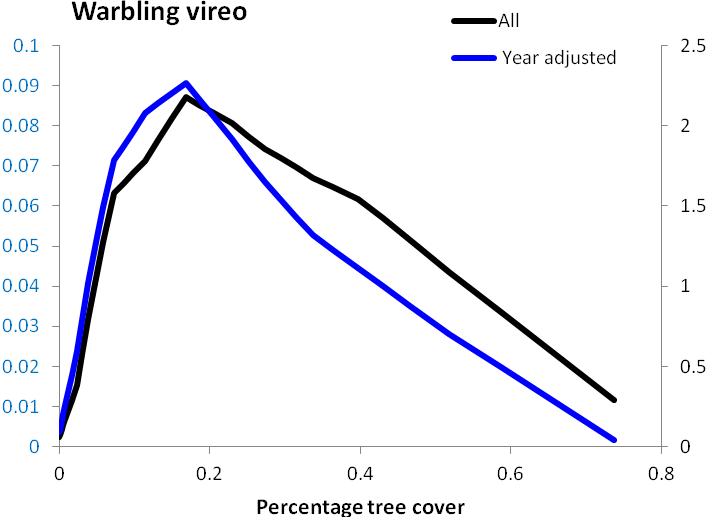


Figure 4 Standard (1/0) and year-adjusted results for four species *evenly* distributed among years


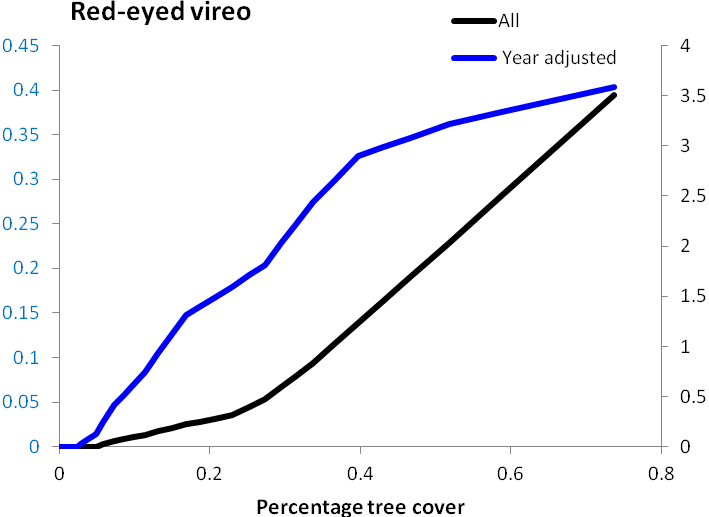

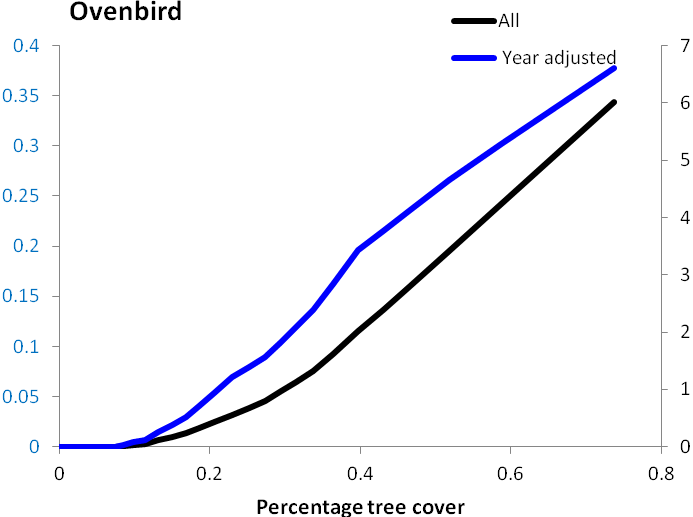

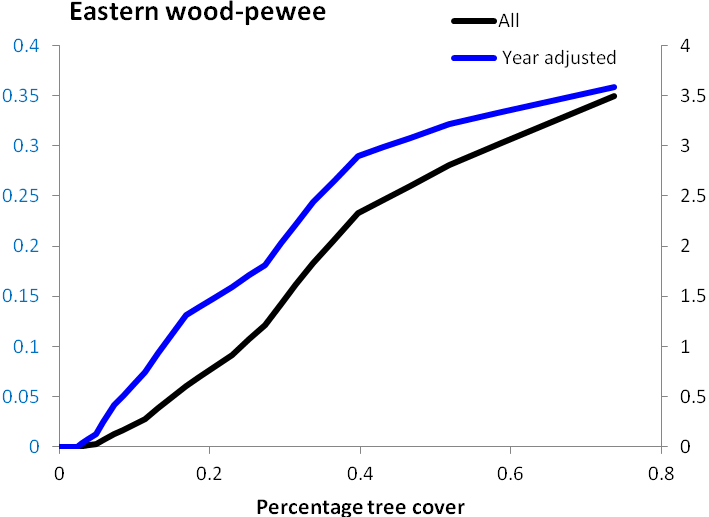

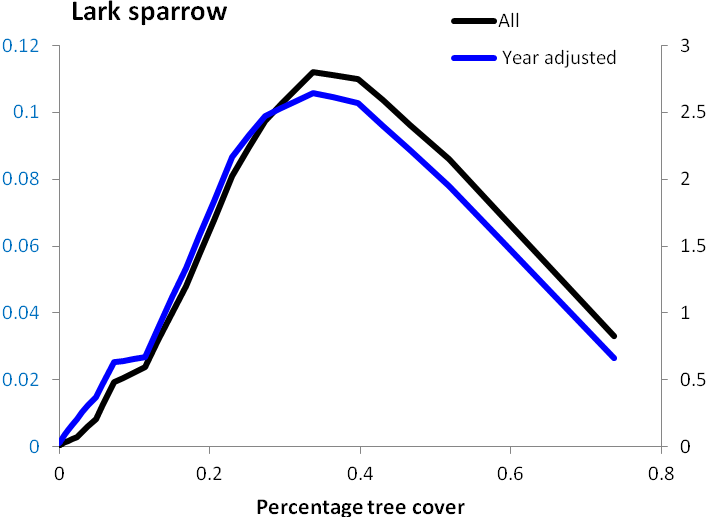


Figure 5 Standard (1/0) and year-adjusted results for four species *unevenly* distributed among years
