# Supplementary material for: Habitat selection and ranges of tolerance: how do species differ beyond critical thresholds?
Source: Ecol Evol. 2012 Oct 9;2(11):2815–28. doi: 10.1002/ece3.394 (PMC3501633; doi:10.1002/ece3.394)

**Appendix S2**. **Results at Three scales**

Incidence patterns showing occurrence in response to proportion tree cover within 200, 400, and 1200 m around transect segments. Dots represent groups of 76 observations; lines show smoothed trends and 95 percent confidence intervals for trends.


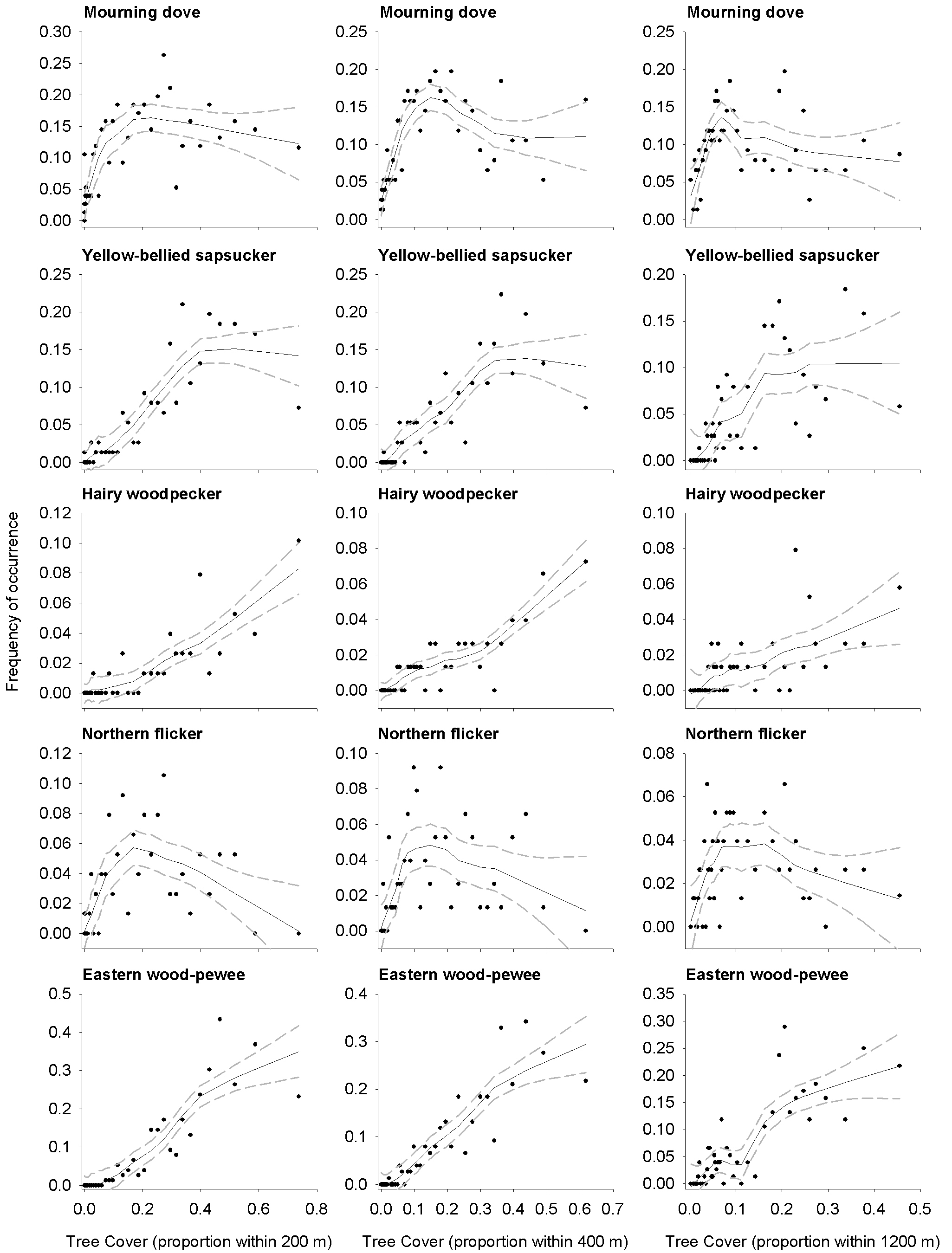


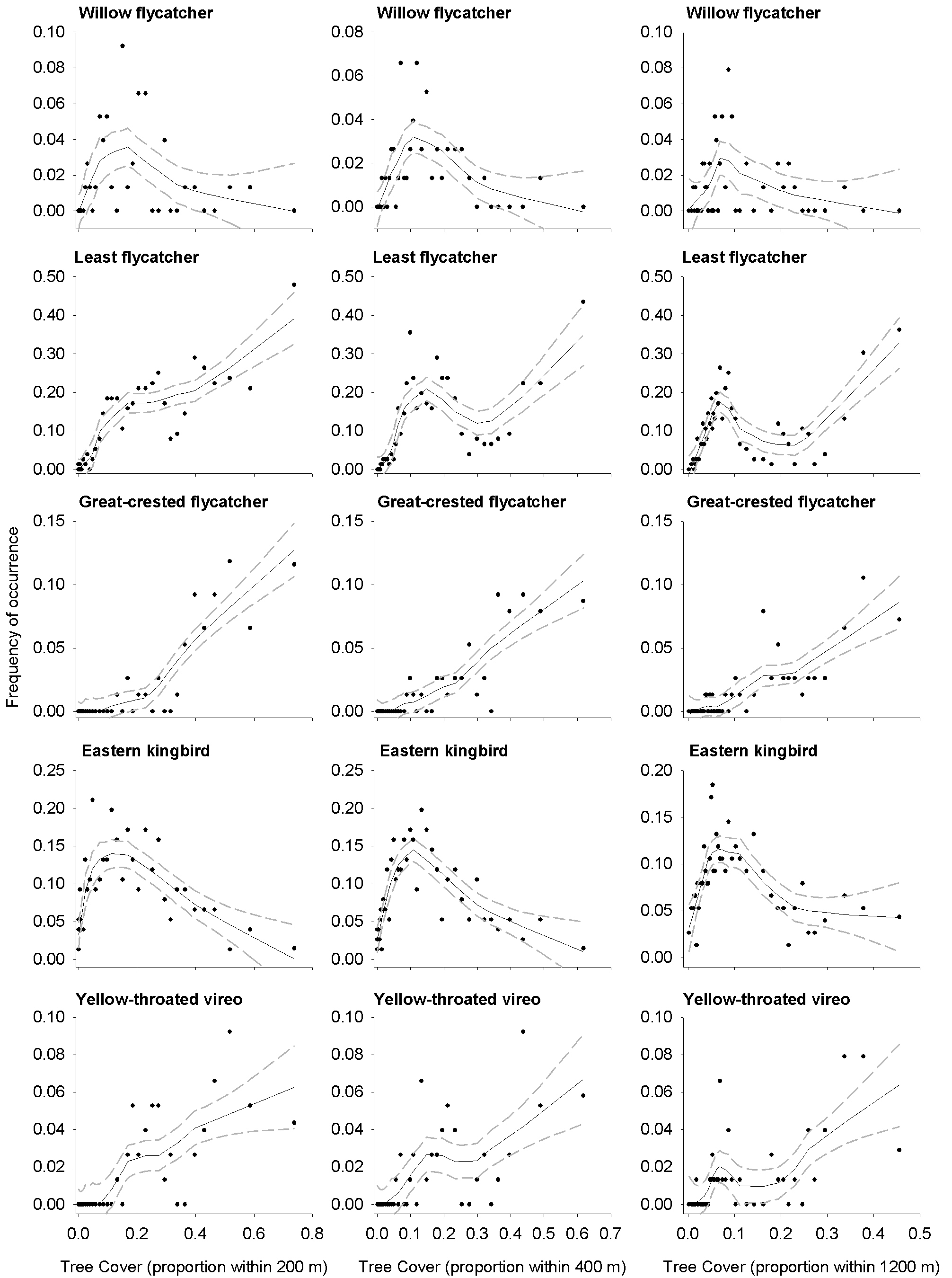


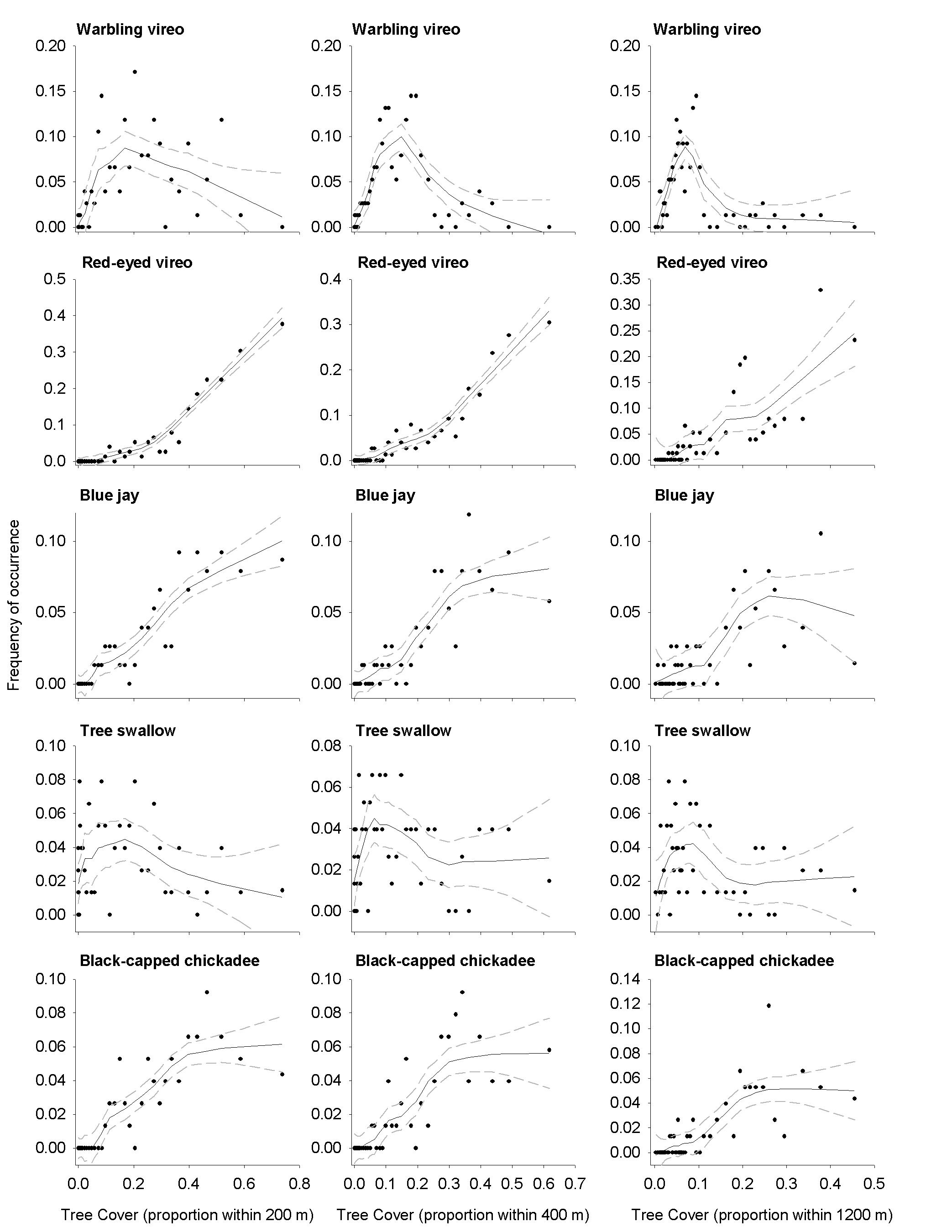


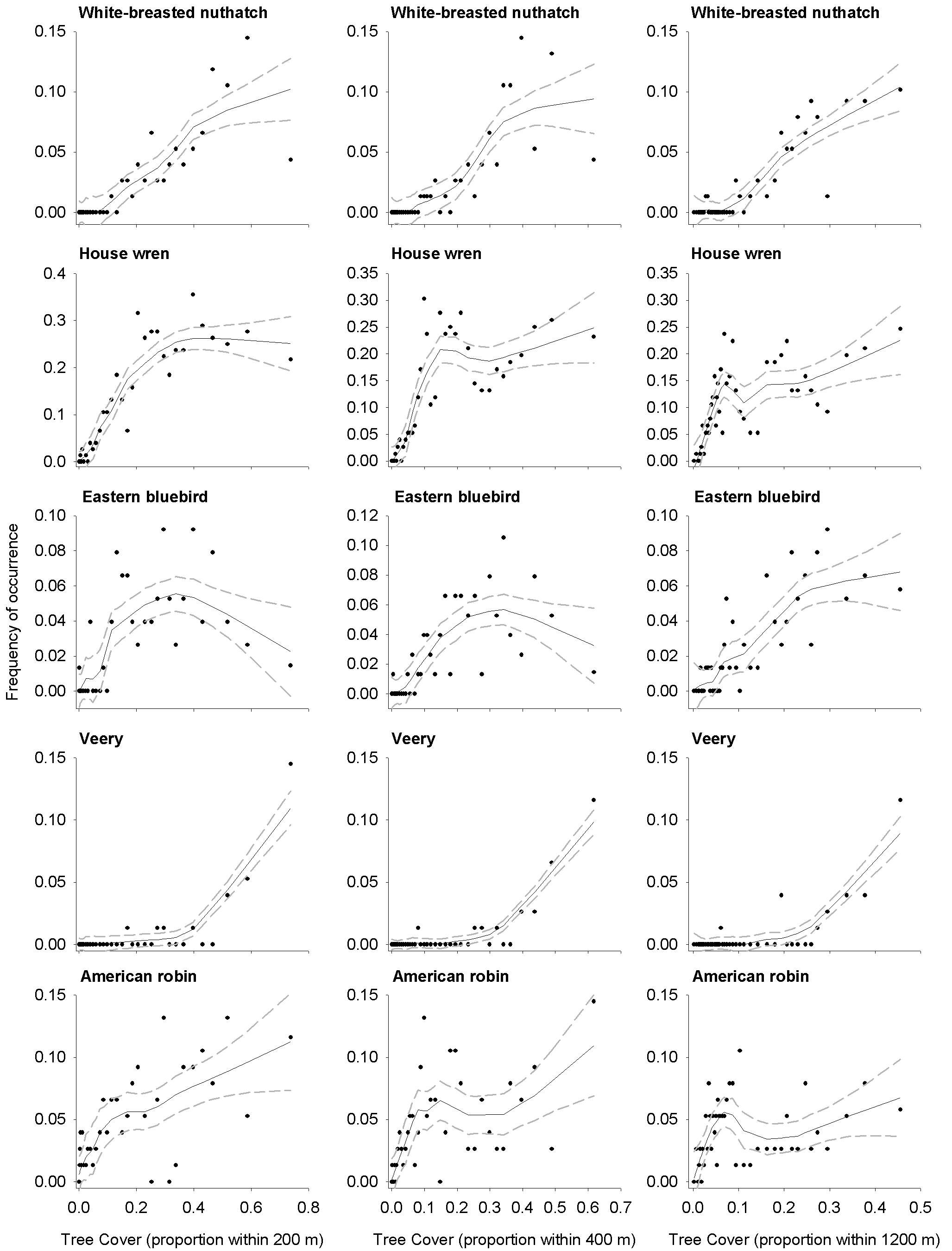


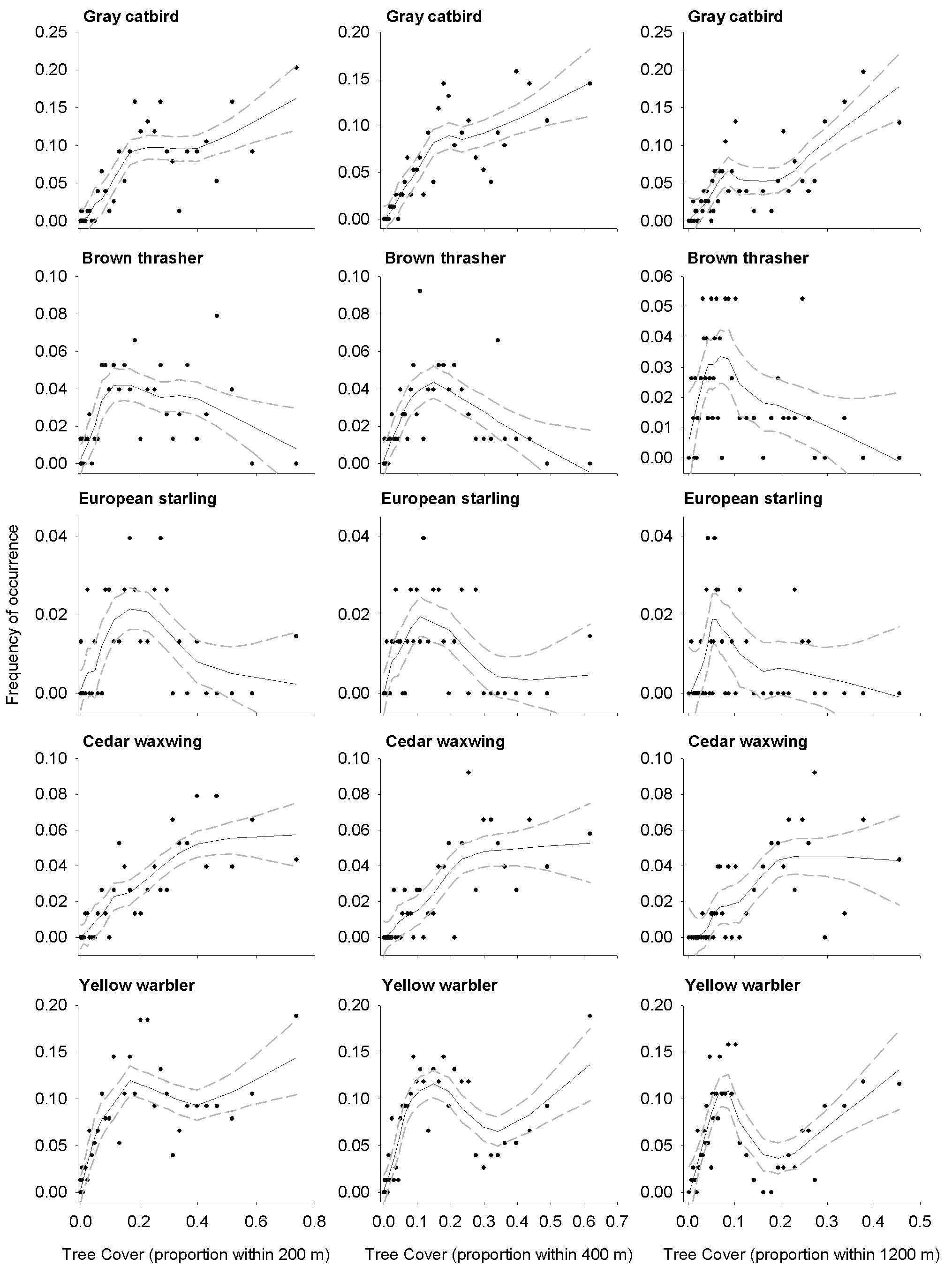


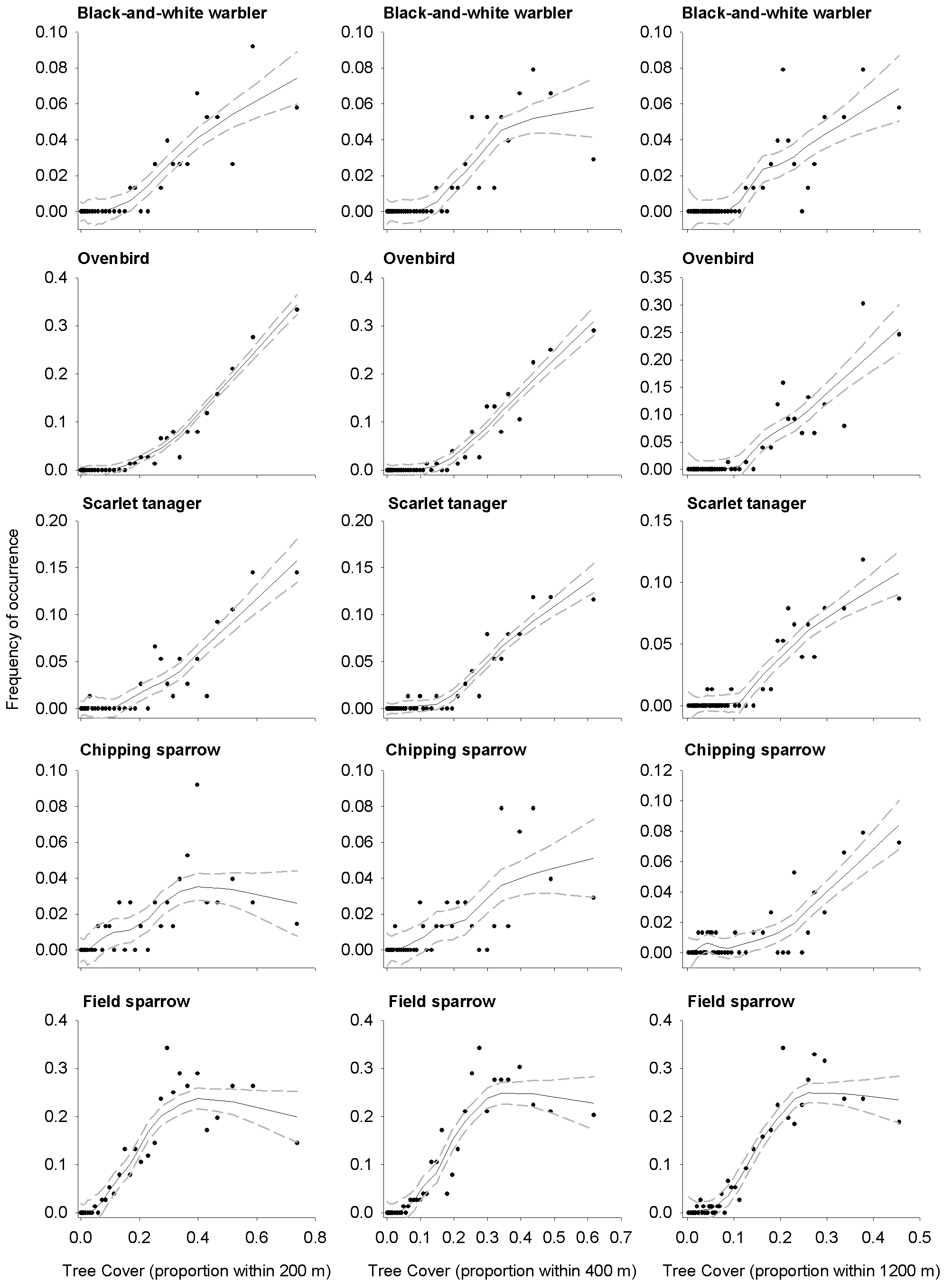


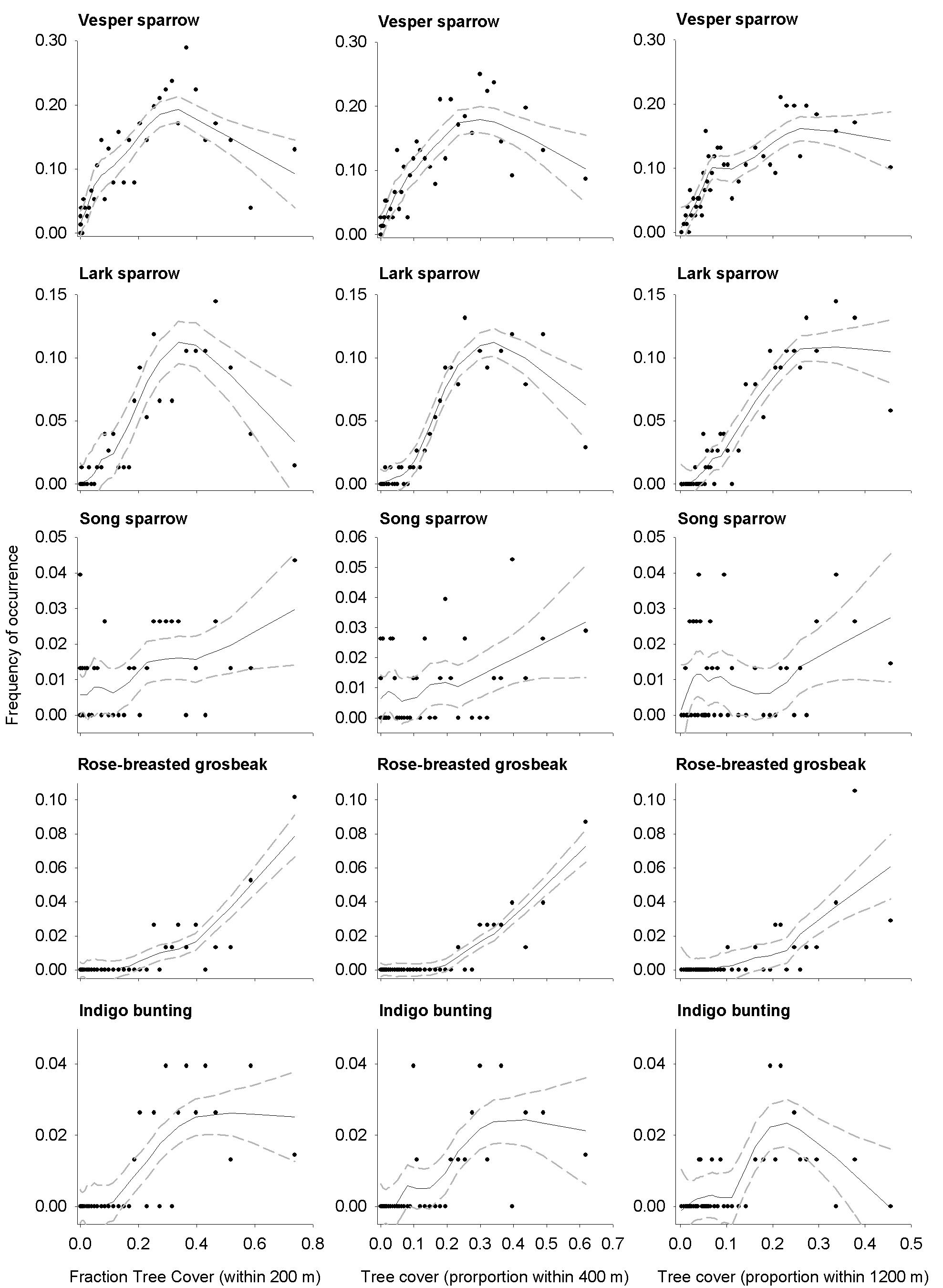


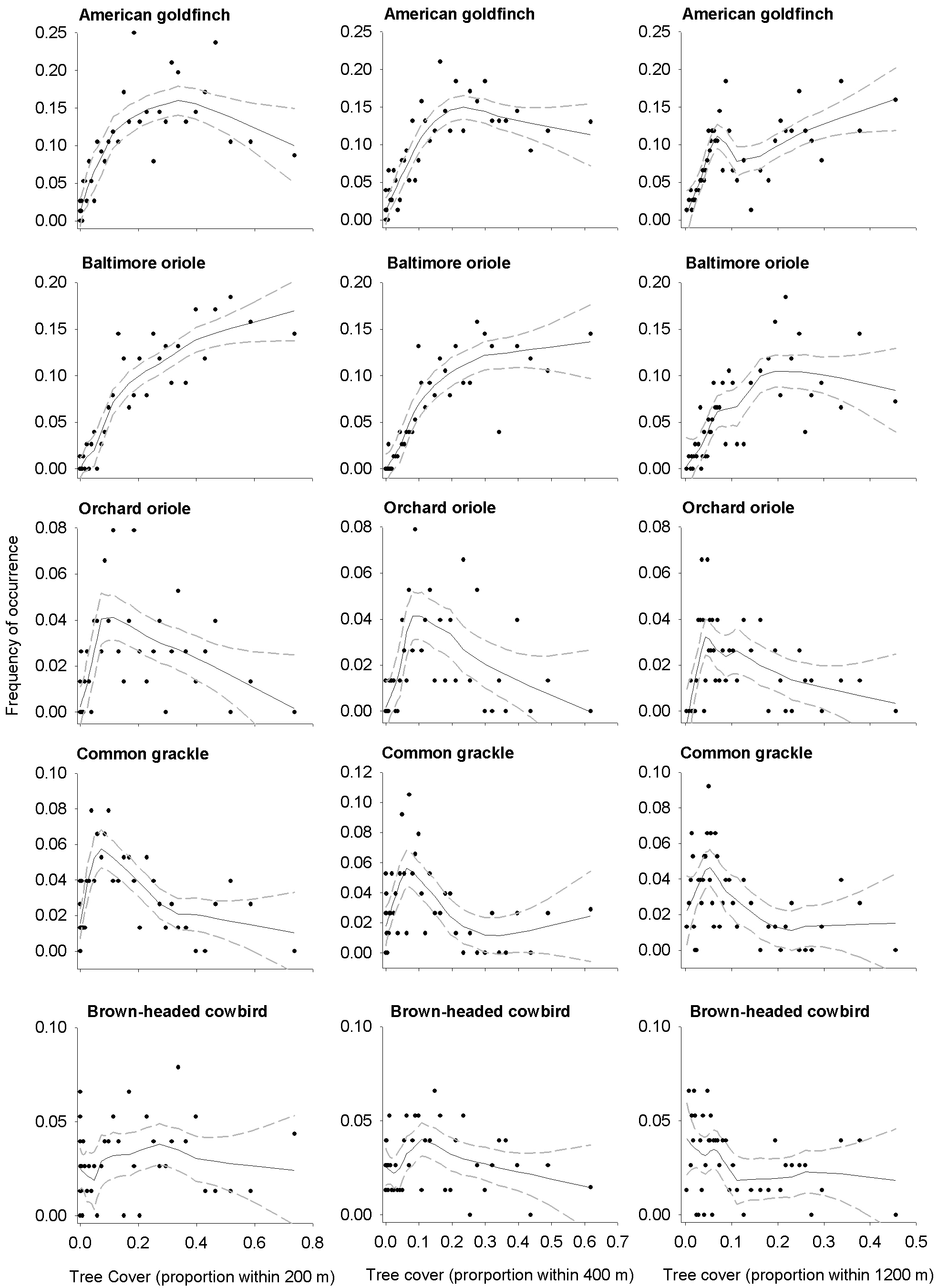

Supplement: Supplementary file 2 [file ece30002-2815-SD2.doc]
